# Mutagenesis of *PhaR*, a Regulator Gene of Polyhydroxyalkanoate Biosynthesis of *Xanthomonas oryzae* pv. *oryzae* Caused Pleiotropic Phenotype Changes

**DOI:** 10.3389/fmicb.2018.03046

**Published:** 2018-12-17

**Authors:** Ju-Ying Long, Kang-Li Song, Xiang He, Bin Zhang, Xiao-Fang Cui, Cong-Feng Song

**Affiliations:** Department of Plant Pathology, College of Plant Protection, Key Laboratory of Integrated Management of Crop Diseases and Pests, Ministry of Education, Nanjing Agricultural University, Nanjing, China

**Keywords:** *Xanthomonas oryzae* pv. *oryzae*, polyhydroxyalkanoate regulator, virulence, motility, hypersensitive response, *hpa1* gene, extracellular polysaccharide

## Abstract

Polyhydroxyalkanoates (PHAs) are intracellular carbon and energy storage materials produced in various microorganisms under nutrient-limited conditions. PhaR is a regulatory protein involved in PHA synthesis. *Xanthomonas oryzae* pv. *oryzae* (Xoo) is one of the most important bacterial pathogens in rice and has PHA biosynthesis genes in its genome, but the biological function of phaR in *Xoo* is unknown. In this study, we investigated the effects of the mutagenesis of *phaR* gene in *Xoo* strain PXO99^A^. Compared to the wildtype, the *PhaR* gene knock-out mutant PXO99ΔphaR was hypermotile and showed decreased growth rates in both rich and limited nutrient media. PXO99ΔphaR also showed almost 75% decrease in extracellular polysaccharide (EPS) production. When inoculated in rice leaves by leaf-clipping method, PXO99ΔphaR displayed reduced virulence in terms of lesion length and bacterial multiplication compared with the wildtype strain. PXO99ΔphaR also showed enhanced hypersensitive response (HR) induction in the leaves of non-host *Nicotiana benthamiana* with elevated *hpa1* gene expression. Introduction of a cosmid containing the phaR coding sequence restored the phenotypes of the mutant to those of the wildtype strain. These results suggest that *PhaR* gene is an important gene that affects multiple bacterial characteristics, including EPS production, growth rate, defense response induced harpin production and motility, related to its virulence in plant.

## Introduction

Bacterial blight on rice caused by *Xanthomonas oryzae* pv. *oryzae* (Xoo) is a widely prevalent disease that causes serious rice production losses worldwide (Singh et al., 1978; Nino-Liu et al., 2006). Xoo invades rice leaf through wounds or natural openings like hydathodes, then colonizes the intercellular space, hijacking the nutrient from host and dispensing along vascular bundles, to cause leaf blight (Mew et al., 1984; Bezrutczyk et al., 2018). During interactions with host rice and non-host, like tobacco, Xoo largely depends on Type III secretion system (T3SS) to secrete different effector proteins to cause disease or induce hypersensitive response (HR) (Wei et al., 1992; Alfano and Collmer, 2004; Song and Yang, 2010). There are also other factors contributing to the virulence of Xoo, including extracellular polysaccharides (EPSs) and plant cell wall degrading enzymes. Typically, motility and bacterial virulence are positively related, but it has been reported that hypermotility could also cause reduced virulence (Ottemann and Miller, 1997; Meng et al., 2011).

Polyhydroxyalkanoates are biodegradable polyesters synthesized by most bacterial genera and some archaea under unbalanced source of carbon or nitrogen (Lee, 1996; Steinbuchel and Fuchtenbusch, 1998; Ciesielski et al., 2010). PHAs are water-insoluble granules stored in the cytoplasm as carbon-storage and energy storage materials, which bacteria degrade and use for energy when faced with starvation. Genes involved in PHA synthesis often form gene clusters on bacterial genomes (Maehara et al., 2001). These genes are cataloged into two groups, one encoding proteins for granule-associated compounds, the other encoding regulators involved in the regulation of structural proteins and PHA biosynthesis. Polyhydroxybutyrate (PHB) is perhaps the most common type of PHAs (Pötter and Steinbuchel, 2005; Rehm and Steinbüchel, 2005). The biosynthesis of PHB starts usually with the production of Acetoacetyl-CoA via the condensation of two molecules of acetyl-CoA catalyzed by a β-ketothiolase (PhaA); acetoacetyl-CoA is subsequently reduced by a stereospecific acetoacetyl-CoA-reductase (PhaB) to R-(-)-3-hydroxybutyryl-CoA, which finally is catalyzed by the PHA synthase and polymerizes the acyl moieties of 3-hydroxybutyryl-CoA to PHB with concomitant release of coenzyme A (Pötter and Steinbuchel, 2005). PhaR is a putative cytoplasmic regulator which can bind to the promoter of phaP, a PHB granule associated protein, and to the promoter of its own gene to repress transcription (Cai et al., 2015). PhaR is conserved in PHA-producing bacteria. In the model PHA-producing strain *Ralstonia eutropha* H16, PhaR functions as a repressor or autoregulator for the expression of PhaP and PhaR itself, both of which can tightly bind to PHB granules (York et al., 2002; Pötter et al., 2005). PHA synthesis related genes also exist in phytopathogenic bacteria, including *Xanthomonas*, but limited research exists on the biological functions of these genes during bacterial growth and host infection. In this study, we demonstrated that loss of *phaR* in Xoo strain PXO99^A^ alters multiple physiological and biological functions which affect bacterial growth and virulence in plant, and provided new insights into the biological function of PhaR in *X. oryzae* pv. *oryzae*.

## Materials and Methods

### Bacterial Strains, Plasmids and Growth Conditions

The bacterial strains and plasmids used in this study are listed in Supplementary Table S1. Xoo strains were grown in either nutrient broth (NB) medium or hrp-inducing medium XOM2 (Xiao et al., 2007) at 28°C. The antibiotics used in this study were spectinomycin (50 μg mL^-1^), kanamycin (25 μg mL^-1^) and ampicillin (100 μg mL^-1^).

### Construction of *PhaR* Knockout Mutant and Mutant Complementation

The *PhaR* gene knockout construct was made by overlap PCR to produce a chimeric DNA including 892 bp upstream flanked DNA of *phaR* gene, 1.4 kb kanamycin cassette from vector pKD13 and 729 bp downstream flanked arm of *phaR* gene. This chimeric DNA was then cloned into pMD19 through T-A cloning. The resulting plasmid was introduced into PXO99^A^ cells by electroporation (Song and Yang, 2010). The single exchange mutant was screened on kanamycin and ampicillin-containing media. The second exchange occurred simultaneously during the culturing in a liquid medium without any antibiotics. The clones that could grow on NA medium supplemented with kanamycin and ampicillin-sensitive were chosen as *phaR* gene deficient mutant candidates for further PCR characterization.

To complement the *phaR* mutant, the cosmid pHMPhaR containing the coding sequence of *phaR* gene was transformed into PXO99ΔPhaR by the freeze-thaw method (Dityatkin et al., 1972) to make the complementary transformant C-ΔphaR. Primers used for construction are listed in Supplementary Table S2.

### Bacterial Motility Assays

For motility assay, bacterial strains were grown in NB medium for 2 days. Bacterial cells were then harvested by centrifugation, re-suspended in sterilized distilled water, and adjusted to an optical density (OD_600_) of 1.0. 1 μL of bacterial suspension was then dropped onto the plates containing semisolid medium (containing per liter 5 g Bacto Peptone, 1 g yeast extract, 3 g beef extract, 10 g sucrose and 0.03 g agar). Plates were incubated at 28°C for 3 days. The diameters of the swimming zones indicated the ability of bacterial movement. The experiment was repeated four times with three replicates for each time.

### Bacterial Extracellular Polysaccharides (EPS) Measurement

Bacterium was cultured in NB overnight at 28°C. An aliquot of 20 mL culture was taken out and added with KCl to a final concentration of 1%. Then two volumes of absolute ethanol were added to precipitate EPS at -20°C overnight. EPS was collected by centrifuging at 12000 rpm for 20 min, and the pellet was dried at 55°C overnight before weighed. The bacterial concentrations of the culture used for EPS extraction were measured by diluting serially and plating in triplicate on NA plates to count the colony numbers. The production of EPS was evaluated in terms of weight (g) per cfu. The experiment was repeated three times.

### Virulence Assay in Rice and Hypersensitive Response in Tobacco

One-month-old rice cv. IR 24 seedlings were used for virulence assays. The top two fully expanded leaves were inoculated with Xoo strains at OD_600_ of 0.5 via the leaf-clipping method. Lesion lengths were measured 15 days after inoculation, and the inoculated leaves were then pooled and cut into small pieces and ground in sterile water (with 1 ml sterile water per leaf) using a sterilized mortar and pestle. The resulting suspension was diluted serially and plated in triplicate on NA agar with appropriate antibiotics. Counts were taken and converted into CFU per leaf.

One-month-old *Nicotiana benthamiana* was used for HR assays. A bacterial suspension at OD_600_ of 0.2 was infiltrated into leaf tissue using a needleless syringe. Symptoms were recorded 24 h after infiltration. The assays for micro-HR response, active oxygen burst, and ionic conductivity of tobacco leaf were performed 12 h after infiltration as previously described (Peng et al., 2003; Fu et al., 2007). For *in situ* detection of H_2_O_2_ at indicated time points, tobacco leaves were detached and vacuum infiltrated in a 3,3′-diaminobenzidene (DAB) solution (1 mg/ml, pH 3.8) for 10 min, and then incubated at 25°C for 7–8 h. The deposits formed were visualized under microscopy after discoloring leaves in boiled absolute ethanol. Micro cell death was monitored by staining with lactophenol–trypan blue and destaining in chloral hydrate (2.5 mg/ml) as described by Peng et al. (2003).

### RNA Isolation and qRT-PCR

Bacteria strains were grown either in nutrient broth (NB) medium or in *hrp*-inducing medium XOM2 overnight at 28°C (Xiao et al., 2007). 1 mL culture was collected for RNA isolation using SV Total RNA Isolation System (Promega, Madison, WI, United States). Reverse transcription was carried out using PrimeScript RT Master Perfect Real Time Kit (TakaRa Bio. Inc., Dalian). Quantitative real-time PCR (qRT-PCR) was performed using SYBR Premix Ex TaqTM Kit (TaKaRa Bio. Inc., Dalian). 16s rDNA was used as internal reference gene. The primer sets used are listed in Supplementary Table S2.

### Phylogenetic Analysis of PhaR Family

Phylogenetic analysis of the aligned full-length sequence of *phaR* gene was conducted with MEGA 3.1 using neighbor joining and the Poisson correction model (Saitou and Nei, 1987; Kumar et al., 2004). Pairwise deletion was used for handling of sequence gaps. Members of the PhaR effector family used for phylogenesis analysis are indicated as their bacterial origins. The responding locus tags and bacterial origins are listed in Supplementary Table S3. The sequences used for phylogenetic tree generation are listed in Supplementary Table S4.

### Data Analysis

For relative gene expression assays, Student’s *t*-test at the 99% confidence level was performed. One-way analysis of variance (ANOVA) statistical analyses were performed on the motility assay, EPS production assay and virulence assay. The Tukey honest significant difference test was used for post-ANOVA pairwise tests for significance, set at 5% (*P* < 0.5).

## Results

### Identification of *phaR* Gene in PXO99 and Its Homologs in Several Phytopathogenic *Xanthomonas* Strains

**FIGURE 1:**
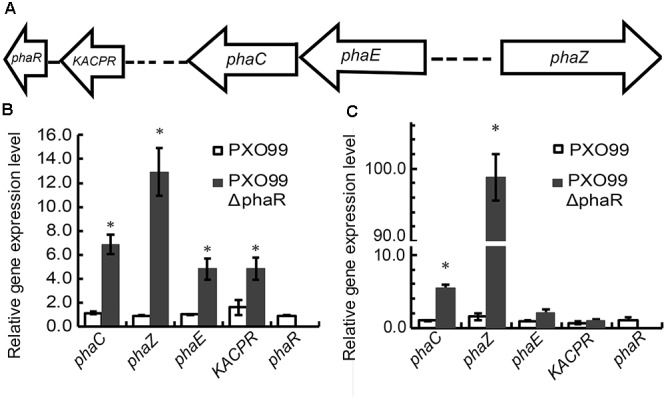
The effect of *phaR* mutagenesis on the expression levels of other genes. **(A)** Genetic organization of the *pha* gene cluster. The arrows indicate the direction of gene transcription. **(B,C)** The relative expression levels of *phaC*, *phaE*, *KACPR*, *phaZ*, and *phaR* in *phaR* mutant and wildtype cultured in NB medium at 12 h **(B)** and 22 h **(C)** respectively, after inoculation. Error bars represent ±SD. The asterisk (^∗^) indicates a significant difference in Student’s *t*-test at *p* < 0.01.

Based on the genomic sequence annotations of Xoo strain PXO99, five PHA metabolism related genes were found (Figure 1A), which include a beta-ketoacyl-ACP reductase (*KACPR*, PXO_RS09075) gene, a polyhydroxyalkanoate synthesis repressor (*phaR*, PXO_RS09070) gene, a poly(R)-hydroxyalkanoic acid synthase subunit PhaC (*phaC*, PXO_ RS11155) gene, a PHA synthase subunit (*phaE*, PXO_RS11160,) gene, and PHB depolymerase (*phaZ*, PXO_RS14020) gene. Among these, *phaC* and *phaE* were adjacent. To investigate the relationship of *phaR* with other related genes, we qualified the transcriptional expression levels of *KACPP*, *phaC*, *phaZ*, and *phaE* genes in the *phaR* mutant and the wildtype. Considering *phaR* gene is a stress responsible gene, we selected two time points, the first at 12 h representing the early stage of growth with the equal growth rates of mutant and wildtype strains, the other at 22 h representing the later stage with differential growth rates of mutant and wildtype strains. The results showed that mutagenesis of *phaR* gene affected the expression of other PHA metabolism related genes (Figures 1B,C). At both stages, the most significantly affected was the expression of *phaZ*, responsible for the degradation of PHB granule. The relative expression levels of *phaZ* for *phaR* mutant were 13.9 and 65.8 folds of those in the wildtype at 12 and 22 h after inoculation, respectively. The expression of *phaC* and *phaE* were also induced in the mutant, with 5.9 and 5.7 folds of induction for *phaC* at 12 and 22 h, respectively, and 4.7 and 2.1 folds of induction for *phaE* at 12 and 22 h, respectively. These results indicate that the bacteria may produce poly(3-hydroxybutyrate) (PHB), a type of PHA, and that *phaR* gene of PXO99 negatively regulated the degradation of PHB, which was more apparent in the late stage of growth with insufficient oxygen and nutrients.

**FIGURE 2:**
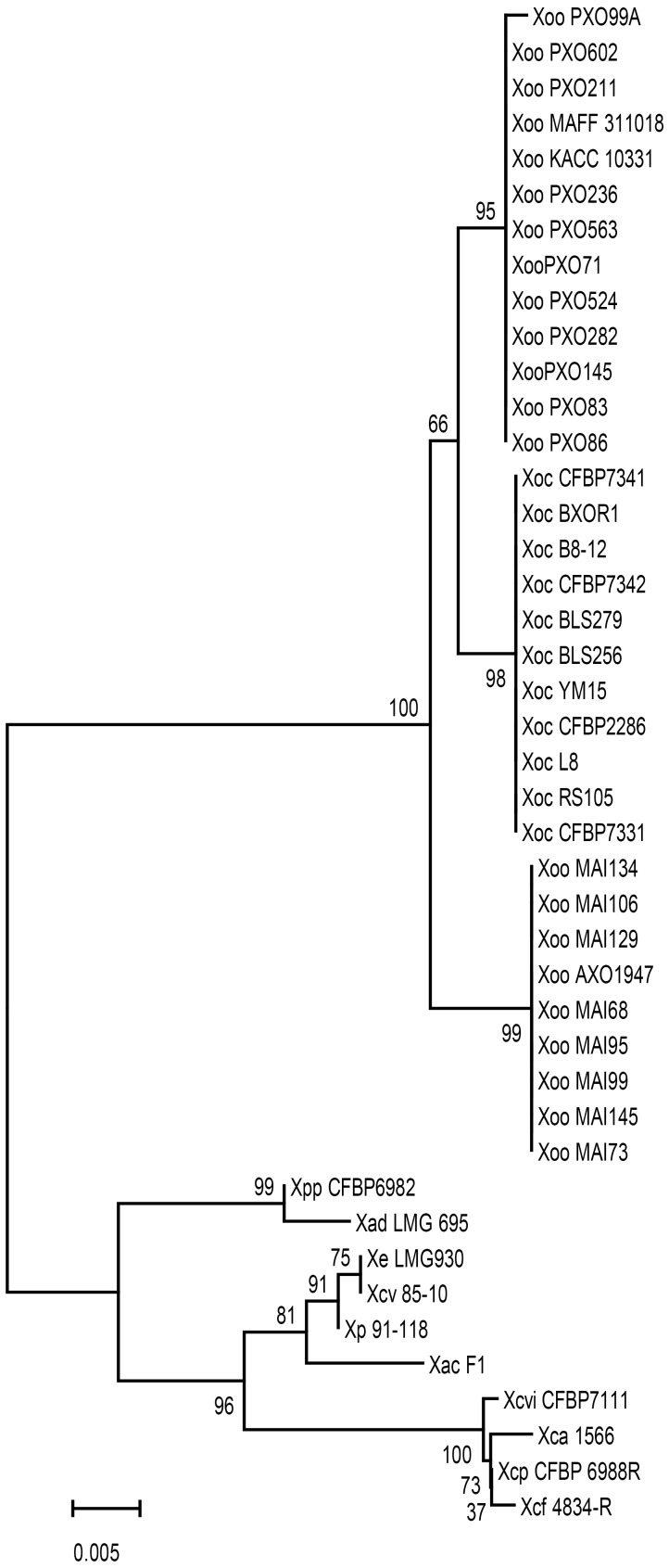
Unrooted phylogenic tree of *phaR* homologs from different *Xanthomonas* species. Bootstrap values shown at nodes were obtained from 1000 trials, and branch lengths correspond to the divergence of sequences, as indicated by the relative scale (20% weighted sequence divergence).

The Xoo *phaR* gene is commonly found in plant pathogenic *Xanthomonas* species, but the gene sequences from different strains are polymorphic. The phylogenetic relationship was established based on *phaR* gene nucleotide sequences of 43 strains of *Xanthomonas* (Figure 2). As shown in Figure 2, the phylogenomic relationship displayed host, tissue, and geographic specificity. There are two main phylogenetic groups. One consists of strains of Xoo and *X. oryzae* pv. *oryzicola* (Xoc), which infect monodicot. The other group, with the exception of *X. axonopodis* pv. *dieffenbachiae* LMG 695 (Xad LMG695), a pathogen of monocot Ariod plants (Robène et al., 2016), consists of dicot plant parasites, including *X. axonopodis* pv. *phaseoli* (XapCFBP6982, pathogen of bean (Gent et al., 2005), *X. perforans* (Xp91-118, pathogen of *Solanacearum* plant (Itako et al., 2015), *X. euvesicatoria* LMG930 (Xe LMG930, pathogen of pepper Vancheva et al., 2018), *X. campestris* pv. *vesicatoria* (Xcv85-10, pathogen of pepper and tomato; Jones et al., 2004; Quelas et al., 2016), *X. axonopodis* pv. *citrumelo* F1 (XacF1, pathogen of citrus Jalan et al., 2011), *X. citri* pv. *vignicola* strain CFBP7111 (XcivCFBP7111, pathogen of cowpea Ruh et al., 2017a), *X. fuscans* subsp. *aurantifolii* strain 1566 (Xfa1566, pathogen of citrus Rigano et al., 2007), *X. phaseoli* pv. *phaseoli* (XppCFBP6988, pathogen of common bean Ruh et al., 2017b), *X. fuscans* subsp. *fuscans* str. 4834-R (Xff4834-R, pathogen of common bean Aritua et al., 2015). Within the first group, 11 Xoc strains infecting the mesophyll tissue formed a clade, and had identical sequences. Interestingly, 22 Xoo strains were divided into two clades, one for 9 African strains with identical sequences, the other for 13 Asian strains originated from Japan, Korea and the Philippines having almost identical sequences except one sequence from PXO99 strain with a 1 bp difference.

### Loss of *phaR* Reduced the Growth Rate and EPS Production in Medium

**FIGURE 3:**
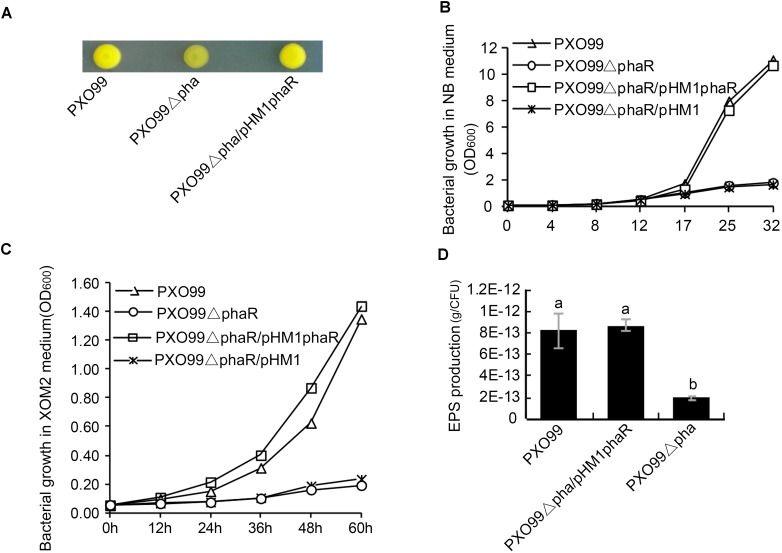
Colony morphology and EPS production of *phaR* mutant. **(A)** Colony morphology on NA plates. 1 μL bacterial suspension with OD_600_ of 0.5 was spotted on NA plate, and the photo was taken after the plate was incubated at 28°C for 3 days. **(B,C)** Bacterial growth in XOM2 **(B)** and NB liquid media **(C)**. The bacterial strains were inoculated with the final concentration of OD_600_ of 0.05, and the OD_600_ of the culture was measured at different time points. The experiment was repeated three times. **(D)** EPS production. EPS from 20 ml bacterial culture was precipitated with ethanol and KCl. The EPS mass was measured after drying the pellet under 65°C for 72 h. The EPS productivity was counted in terms of g/cell. Error bars represent ±SD. Columns with different letters represent significant difference by ANOVA (*p* < 0.05).

To examine the effects of *phaR* mutagenesis on the morphology of PXO99^A^, we spotted the wildtype PXO99^A^, the mutant PXO99ΔphaR and the complemented strain C-ΔphaR on NA plates. 3 days later, PXO99ΔphaR developed dry colonies with reduced slime on the surface, while PXO99^A^ and C-ΔphaR had normal mucoid colonies (Figure 3A). This indicated that *phaR* gene might regulate the growth rate of the bacteria or affect the production of EPS.

To test if *phaR* regulates bacterial growth, we examined the growth curves of *phaR* mutant both in rich (NB) and minimal (XOM2) nutrient medium. The results showed that, in both media, the growth rate of PXO99ΔphaR was reduced greatly compared with that of the wildtype (Figures 3B,C). When inoculated with the same starting concentrations in liquid NB and XOM2 medium, the wildtype PXO99^A^, mutant PXO99ΔphaR and the complementary strain PXO99ΔphaR/phaR all grew at the similar rate for the first few hours, and then started to differ about 12 h after inoculation. PXO99ΔphaR kept propagating steadily but at much lower level, with the population size about 15.9 and 14.1% of the wildtype at 32 h (in NB) and 60 h (in XOM2), respectively (Figures 3B,C). The complement strain grew similarly as the wildtype in both media.

Since PXO99ΔphaR displayed the dry colony phenotype, we next tested if the EPS production was affected in the mutant. To do this, we extracted and compared the EPS mass from the 46 h NB culture supernatant of PXO99^A^ and PXO99ΔphaR. With the same volume of culture supernatant, significantly less EPS pellet was precipitated by ethanol from the *phaR* mutant than the wildtype. Since the growth of the mutant was reduced compared to the wildtype and the complement strain, we measured the bacterial numbers and determined the EPS production per cell, in order to deduce if the reduced EPS production was caused by a reduced amount of bacteria or by the reduced EPS production efficiency. The results showed that EPS productivity of the mutant was greatly impaired and was about 22% of the wildtype (Figure 3D). This suggests that *phaR* positively regulates EPS production in PXO99^A^.

### Loss of *PhaR* Reduced the Multiplication of PXO99^A^ in Rice

**FIGURE 4:**
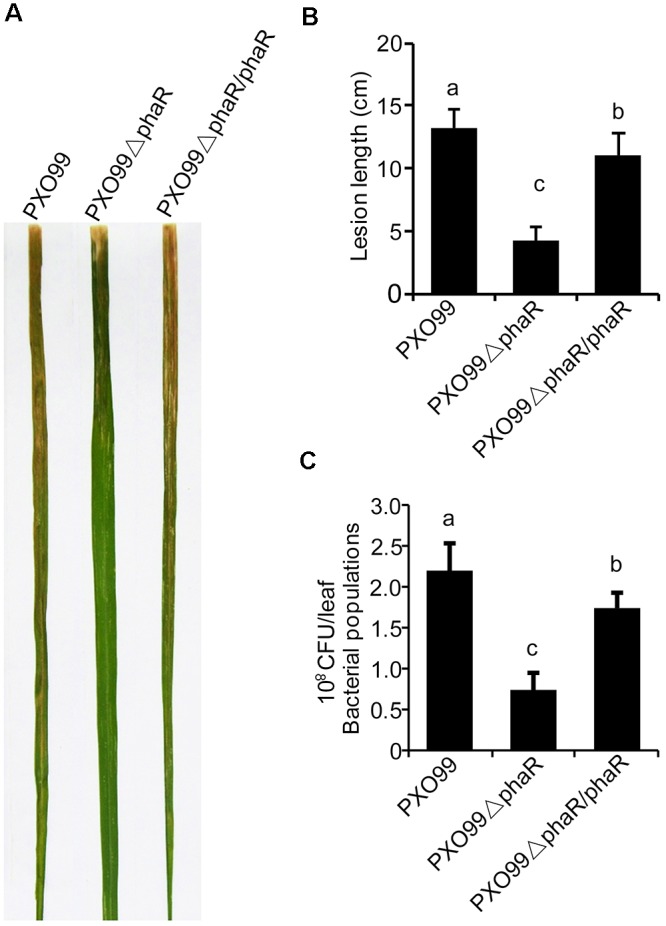
*PhaR* mutant had reduced virulence in rice. **(A)** Lesions formed in rice IR24 leaves 14 days after leaf-clipping inoculation with different strains. **(B)** Lesion lengths caused by different strains 14 days post-inoculation. **(C)** Bacterial population sizes of different strains in rice. Error bars represent ±SD. Columns with different letters above were significantly different by ANOVA (*p* < 0.05).

Since *phaR* mutagenesis affected the bacterial growth in media, esp. in XOM2, a medium mimicking the host nutrient state *in vitro*, we hypothesized that it might also affect the bacterial propagation of Xoo in rice. To test this, we inoculated rice cv. IR24 with PXO99^A^, PXO99ΔphaR, and PXO99ΔphaR/ΔphaR by the leaf-clipping method. 14 days after inoculation, the average lesion length caused by PXO99ΔphaR inoculation was about 4 cm, which was about one third of that caused by wildtype strain PXO99^A^ (Figures 4A,B). The bacterial multiplication in the inoculated leaves was measured by counting the colonies grown on media. Compared with PXO99^A^, the population size of PXO99ΔphaR in rice was reduced about 68% (Figure 4C). Complementation of the mutant with *phaR* gene restored the phenotype similar to the wildtype level. This data showed that the growth of PXO99ΔphaR is drastically restrained due to the loss of the *phaR* gene, which then reduces the overall virulence of Xoo in rice.

### PXO99ΔphaR Mutant Was Hypermotile

**FIGURE 5:**
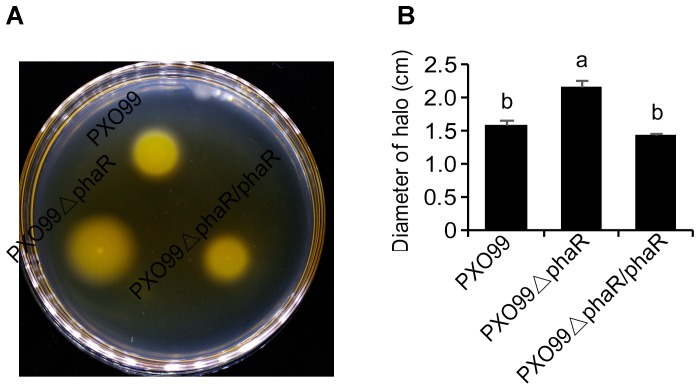
Motility phenotypes of different strains. **(A)** Typical bacterial halos, formed on semisolid 0.3% agar plates after incubation at 28°C for 3 days. **(B)** Diameters of motility halos, formed on semisolid 0.3% agar plates. Error bars represent ±SD. Columns with different letters above were significantly different by ANOVA (*p* < 0.05).

The fact that the *phaR* mutant failed to travel to long distance to cause leaf lesion suggested that *phaR* might be involved in bacterial movement. To test this hypothesis, we evaluated the swimming motility of Xoo strains in semisolid motility agar. Unexpectedly, the results showed that PXO99ΔphaR was hypermotile, producing swimming haloes 1.36-fold in diameter of those of the wildtype PXO99^A^. The complementary strain restored motility to wildtype level (Figure 5). Thus, *phaR* might negatively affects motility in PXO99^A^.

### Loss of *PhaR* Enhanced the Elicitation of HR in Tobacco

**FIGURE 6:**
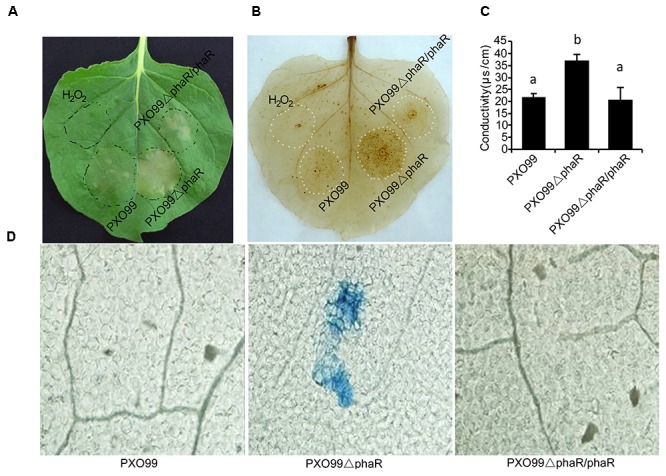
Loss of PhaR increased HR, H_2_O_2_ accumulation and ion leakage in leaves of *Nicotiana benthamiana*. **(A)** Macroscopic views of HR. Leaves of 30-day-old tobacco plants were infiltrated with indicated bacterial suspensions. Photos were taken at 24 h post-treatment (hpt). **(B)** ROS detection using the DAB infiltration method. Tobacco leaves infiltrated with bacterial suspension were detached and infiltrated with DAB (1 mg/mL, pH 3.8) by vacuum pump and decoloration. Deposits formed were visualized after discoloring leaves in boiled absolute ethanol. **(C)** Binocular microscope views of micro-HR in tobacco leaves. Leaves of 30-day old tobacco plants were infiltrated with water or indicated bacterial suspension. Leaves were excised at 12 hpt and stained with trypan blue, which stains dead cells blue. **(D)** Histograms of ionic conductivity, which is an indicator of ionic leakage. Ionic conductivity was determined 12 h after tobacco was infiltrated with bacterial suspension. Columns with different letters above were significantly different by ANOVA (*p* < 0.05). Error bars represent ±SD.

In Xoo, pathogenesis on host plants and HR on non-host plants are correlated properties regulated by the *hrp* genes. Because PXO99ΔphaR displayed attenuated virulence in the host plant rice, we next tested if the HR on non-host tobacco would be changed. To do this, a bacterial suspension was infiltrated into tobacco leaves to compare the ability of different strains to induce HR. 24 h after infiltration, PXO99ΔphaR induced an intensive HR and produced a larger necrosis area than that caused by PXO99^A^ (Figure 6A).

To further measure the HR reaction strength, two landmark events of HR, the oxidative burst and micro-HR cell death were examined at 12 hpi through directly staining the biochemical component H_2_O_2_ with DAB or the dead cells with the trypan blue. DAB is oxidized by hydrogen peroxide to give a dark-brown color, and trypan blue selectively stains dead tissues or cells. The DAB staining results showed that PXO99ΔphaR induced extensive and intensive oxidative burst (dark brown spots) around the infection site (Figure 6B). Trypan blue staining of the leaf disks showed PXO99ΔphaR inoculation induced obvious cell death (blue spots), indicating micro-HR occurred around the infiltration site; while for PXO99^A^ treatment, no obvious cell death was found (Figure 6D). Loss of *phaR* gene also increased the ability of PXO99^A^ to induce cell membrane permeability of tobacco (Figure 6C). The reintroduction of the *phaR* gene restored the mutant’s phenotypes similar to those of the wildtype strain (Figure 6). These results indicated that the *phaR* gene negatively regulated HR induction in tobacco.

### Loss of *PhaR* Gene Increased the Expression of Harpin-Encoding Gene *Hpa1*

**FIGURE 7:**
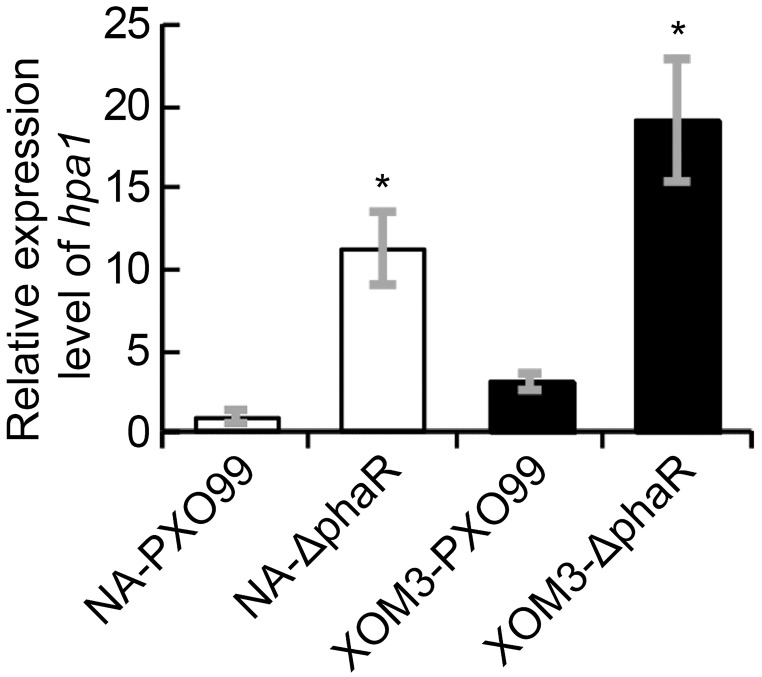
The transcriptional levels of *hpa1* in XOM2 and NB liquid media. The *hpa1* gene expression was measured by quantitative real time PCR and compared between wildtype strain PXO99^A^ and the mutant ΔphaR. The asterisk (^∗^) indicates a significant difference in Student’s *t*-test at *p* < 0.01. Error bars represent ±SD from three replicates.

In Xoo, HR induction in non-host plants is associated with *hrp* genes, esp. the *hpa1* gene which encodes HR inducing harpin protein. To evaluate if the increased induction of HR by PXO99ΔphaR in tobacco is related to the changes of harpin expression, we analyzed the expression levels of *hpa1* gene in different strains under *hrp* inducing medium via quantitative real time PCR (qPCR). The results showed that when cultured in XOM2 medium, which mimics the nutrient state of the plant, the expression levels of *hpa1* in both mutant and wildtype strains increased significantly compared with those in nutrient rich medium NA broth. Moreover, compared with wildtype, the expression of *hpa1* increased significantly in PXO99ΔphaR both in NA and XOM2 broth media (Figure 7). These results confirmed that *hpa1* gene is induced by plant and suggested that *phaR* gene negatively regulated the expression of *hpa1* in Xoo.

## Discussion

Polyhydroxyalkanoates serve as energy and carbon storage compounds under starvation conditions (Anderson and Dawes, 1990). The existence of PHAs metabolism enables the bacterial survival adaptation under poor nutritional condition. In the genome database of PXO99, we analyzed the gene cluster for PHA biosynthesis and metabolism. Searching for *phaA* and *phaB* homolog sequences by BLASTn showed no significant similarity, which indicated that there were no homologs of *phaA* and *phaB* in PXO99. However, adjacent to *phaR*, there is a gene encoding beta-ketoacyl-ACP reductase catalyzing the conversion of 3-hydroxyacyl-CoA to 3-oxyacyl-CoA. Similar as in other PHB producing bacteria, there are *phaC* and *phaE* genes responsible for the biosynthesis of PHB, *phaR* gene for regulation and *phaZ* for PHB degradation in PXO99. The above different compositions of PHB metabolism suggest that there might be different pathways to start the PHB synthesis because of the different nutritional provisions between pathogenic and non-pathogenic bacteria. More interestingly, the phylogenetic analysis based on *phaR* gene sequences suggests that, even though *phaR* gene is widely conserved in phytopathogenic genera of *Xanthomonas*, the polymorphism of nucleotide acid reflects the host specificity and even tissue and geographic region specificity. Bogdanove et al. (2011) previously identified hundreds of tissue and host specificity genes from different completely sequenced *Xanthomonas* strains, but all of these candidate genes were unique genes expressed in strains showing host or tissue infection specificity. However, *PhaR* is a conserved microbe nutrition related metabolism regulator encoding gene in *Xanthomonas* strains. The phylogenetic relationship of *phaR* gene in different phytopathogenic *Xanthomonas* strains displayed host and tissue specificity (Figure 2). This suggests that it may be correlated with the bacterial nutritional acquisition nature for bacteria infecting monocot or dicot plants, and for monocots, the *phaR* gene may be related with different host survival environments. For example, water supply and temperature for rice cultivated in Africa and Asia are very different. In the nitrogen-fixing bacterium *Bradyrhizobium diazoefficiens*, phaR was found to be localized at a higher hierarchy of a global regulator which activates the expression of more than 200 genes needed for bacterial life under low-oxygen tension (Quelas et al., 2016). Although the *phaR* gene is commonly found in different phytopathogenic *Xanthomonas* spp. (Figure 2), the way *phaR* affects bacterial traits related to its pathogenesis in *Xanthomonas* spp. is yet to be explored.

In this study, we made a *phaR* gene knock-out mutant in Xoo strain PXO99 using marker exchange method. By comparing the *phaR* mutant with the wildtype, we discovered that the *phaR* gene is involved in multiple bacterial traits related with bacterial physiology and its pathogenicity on host plants.

Bacterial motility is advantageous as it supports movement toward the host to obtain more nutrients and to avoid toxic environments. Thus, swimming ability is an important virulence factor for phytopathogenic bacteria, such as *Erwinia* spp., *Ralstonia* spp., *Pseudomonas* spp., and *Xanthomonas* spp (Meng et al., 2011; Pique et al., 2015; Kan et al., 2018; Markel et al., 2018). But in this study we found that loss of *phaR* increased the motility of Xoo, and the hypermotile *phaR* mutant displayed decreased virulence in rice. The hypermotility of bacteria may be caused by more flagella or speedy bacterial distribution under irregular environment. While having more flagella may promote the movement of bacteria in the environment, it may also produce abundant flagellin triggering the basal defense response of host. A previous study on *Erwinia amylovora* showed that *E. amylovora* lost motility under starvation condition with no change of flagella (Santander et al., 2014). Also the limited nutrient state *in planta*, XOM2 medium, and xylem sap induced genes related to motility (Kumar Verma et al., 2018). The global regulator *CsrA* gene of the carbon storage constituent of *Escherichia coli* was identified to positively regulate bacterial motility (Wei et al., 2001). The two-component regulatory system response regulator gene mutation studies of *X. oryzae* pv. *oryzicola* showed that, various mutants had similar tendency in EPS production and swarming ability, either increasing together or reducing together, but there was one mutant of *GX_XOC2013* showed decreased EPS production and enhanced swarming ability and significantly reduced virulence on rice (Zhang et al., 2018). We thus speculated that the intracellular nutrient condition altered by the loss of *phaR* affected the flagella’s function. However, the exact regulatory mechanism remains unknown.

Bacterial leaf blight caused by Xoo is a typical vascular disease of rice. For vascular pathogen, EPS plays an important role in virulence. EPS promotes bacterial survival and colonization *in planta* by protecting the pathogen from environmental stresses and host defenses (Buttner and Bonas, 2010; Wu et al., 2012). The *phaR* mutant of PXO99 displayed reduced EPS production and altered colony phenotype as well as an attenuated growth rate. PhaR is a regulator of PHAs metabolism, its dysfunction greatly promoted the expression of *phaZ* gene, increasing the degradation of PHB in PXO99 (Figure 1B), which may affect the biosynthesis of EPS. It was reported that in *Sinorhizobium meliloti* Rm1021, the PHB synthase knockout mutants showed reduced production of the EPS succinoglycan (Aneja et al., 2004). Contrary to our findings, in the nitrogen fixing bacteria *B. diazoefficiens* and *Ensifer meliloti*, the *phaR* mutants produced more EPS (Encarnacion et al., 2002; Quelas et al., 2016). However, the PHB biosynthesis systems of *Ensifer* and *Bradyrhizobium* are quite different in terms of composition from that of PXO99, which suggested that there were different regulation models of *phaR*. For other bacteria, reduced EPS production affected the formation of biofilms, exhibited reduced growth and survival rates on leaf surfaces and reduced disease symptoms (Rigano et al., 2007), or impaired survival under oxidative stress (Dunger et al., 2007). For Xoo, according to the above results, the *phaR* mutant displayed reduced EPS production and relatively low multiplication ability both in nutrient-rich NB or nutrient-limited XOM2 media, or *in vivo* of plant host (Figures 3B,C, 4C), which may result in reduced virulence in rice.

Our experiments also revealed that the *phaR* mutant elicited increased plant defense resistance. Specifically, it induced a strong HR reaction when infiltrated into non-host tobacco leaf. For Xoo, the main effector inducing HR in non-hosts is a proteinaceous elicitor, harpin protein. Harpins are multifunctional and function not only as a part of translocator complexes for effector translocation into plant cells to promote bacterial virulence, but also as inducers of defense responses and as elicitors of HR cell death in the apoplast of plant tissues (Choi et al., 2013). The expression of harpin was greatly increased in the *phaR* mutant compared with the wildtype. The enhanced harpin expression caused by the loss of *phaR* may result from a physiological inability to respond to nutrients. The expression of the harpin protein in Xoo strains is highly related to the nutrition condition that the strain is exposed to Xiao et al. (2007). When the strain is cultured in nutrient-rich medium such as beef broth medium, the expression of harpin protein is suppressed, whereas the expression of harpin protein is induced when there is an interaction between the pathogen and host plant or when pathogen grows in a nutrient-deprived medium, such as XOM2. When PhaR is unfunctional in Xoo, it will reduce the carbon and energy storage by restraining PHA synthesis, or degrading PHB granules, which will in turn cause a state of nutrient deficiency and induce the expression of type III effectors including harpin protein. Our results showed that in both XOM2 and NB media, expression of harpin was induced for *phaR* knock-out mutant (Figure 4). This data indicates that PhaR is a negative regulator for harpin expression. Additionally, the enhanced harpin protein expression maybe a major contributing factor of the *phaR* mutant with enhanced HR inducing ability.

Carbohydrates are one kind of major nutrient sources for Xoo, impairing their corresponding metabolic pathway will result in abnormal bacterial phenotype. In this research we characterized a *phaR* mutant showing impaired EPS production which may reduce the environment viscosity to result in increased motility.

The poor survival adaption indicated by reduced growth rates in both medium and plant as well as increased defense response induced by high expression of harpin and reduced virulence factor EPS production likely resulted in attenuated virulence in rice. Our data suggests that *phaR* is a key gene coupling multiple bacterial characteristics related with its virulence in plant through regulation of carbohydrate metabolism.

## Author Contributions

C-FS and J-YL conceived the study. K-LS, XH, BZ, and X-FC performed the experiments. J-YL and K-LS analyzed the data. C-FS and J-YL wrote the manuscript.

## Conflict of Interest Statement

The authors declare that the research was conducted in the absence of any commercial or financial relationships that could be construed as a potential conflict of interest.
