# Supplementary material for: Mutagenesis of PhaR, a Regulator Gene of Polyhydroxyalkanoate Biosynthesis of Xanthomonas oryzae pv. oryzae Caused Pleiotropic Phenotype Changes
Source: Front Microbiol. 2018 Dec 17;9:3046. doi: 10.3389/fmicb.2018.03046 (PMC6304360; doi:10.3389/fmicb.2018.03046)
Supplement: Supplementary file 1 [file Table_1.DOC]

Supplementary sequences for phylogenetic analysis

>XooPXO99A

TTGGCTGTTGCAAGCGCTGCTGCGGTGCGCCATCCTGCGCGCACTATCAGTGTGGGTGAACGTTCCATGGCCGGACTTCGCATCATCAAGAAGTATCCCAATCGCCGTCTCTACGACACGGAAATCTCCAGCTATATCACCATCGAAGATGTGCGCCAATTGATCATCGATGGCGAAGAATTCGAGGTACGCGATGCCAAGAGTGGCGAAGACCTGAGCCGCGCGGTGCTGCTGCAAATCATCGCCGACCGCGAACAGGACGGCGAGCCGATGCTCTCCACCCAGCTGCTGAGCCAGATCATCCGGTTCTATGGCGATTCGCTGCAGGGCTTCATGGGCAACTACCTGGAGCGCAGCATGCAGGTGTTCCTGGATCAGCAGCAGCAGTTCCGCCAGCAGATGGGCAACCTGCTCGGGCAGACGCCATGGGCGATGATGAACCAGCTGACCGAGCGCAACCTGGAGTTGTGGCAGGAGTTCCAGCGCAATTTCGGCGCCGGCTTCGGTCGCCCGGTTGGCCCTGGCACGCCGGCGAACCCGCCCGGTGCAAGCGGCCTGGGCAGCGGCCCGATTGGAACCGGCACGCACGGCACGCCGAACAATCATCACGGCACCACCGGCAAGGCCCGCAACCGCGGCTAA

>XooPXO602

TTGGCTGTTGCAAGCGCTGCTGCGGTGCGCCATCCTGCGCGCACTATCAGTGTGGGTGAACGTTCCATGGCCGGACTTCGCATCATCAAGAAGTATCCCAATCGCCGTCTCTACGACACGGAAATCTCCAGCTATATCACCATCGAAGATGTGCGCCAATTGATCATCGATGGCGAAGAATTCGAGGTACGCGATGCCAAGAGTGGCGAAGACCTGAGCCGCGCGGTGCTGCTGCAAATCATCGCCGACCGCGAACAGGACGGCGAGCCGATGCTCTCCACCCAGCTGCTGAGCCAGATCATCCGGTTCTATGGCGATTCGCTGCAGGGCTTCATGGGCAACTACCTGGAGCGCAGCATGCAGGTGTTCCTGGATCAGCAGCAGCAGTTCCGCCAGCAGATGGGCAACCTGCTCGGGCAGACGCCATGGGCGATGATGAACCAGCTGACCGAGCGCAACCTGGAGTTGTGGCAGGAGTTCCAGCGCAATTTCGGCGCCGGCTTCGGTCGCCCGGTTGGCCCTGGCACGCCGCCGAACCCGCCCGGTGCAAGCGGCCTGGGCAGCGGCCCGATTGGAACCGGCACGCACGGCACGCCGAACAATCATCACGGCACCACCGGCAAGGCCCGCAACCGCGGCTAA

> XooPXO563

TTGGCTGTTGCAAGCGCTGCTGCGGTGCGCCATCCTGCGCGCACTATCAGTGTGGGTGAACGTTCCATGGCCGGACTTCGCATCATCAAGAAGTATCCCAATCGCCGTCTCTACGACACGGAAATCTCCAGCTATATCACCATCGAAGATGTGCGCCAATTGATCATCGATGGCGAAGAATTCGAGGTACGCGATGCCAAGAGTGGCGAAGACCTGAGCCGCGCGGTGCTGCTGCAAATCATCGCCGACCGCGAACAGGACGGCGAGCCGATGCTCTCCACCCAGCTGCTGAGCCAGATCATCCGGTTCTATGGCGATTCGCTGCAGGGCTTCATGGGCAACTACCTGGAGCGCAGCATGCAGGTGTTCCTGGATCAGCAGCAGCAGTTCCGCCAGCAGATGGGCAACCTGCTCGGGCAGACGCCATGGGCGATGATGAACCAGCTGACCGAGCGCAACCTGGAGTTGTGGCAGGAGTTCCAGCGCAATTTCGGCGCCGGCTTCGGTCGCCCGGTTGGCCCTGGCACGCCGCCGAACCCGCCCGGTGCAAGCGGCCTGGGCAGCGGCCCGATTGGAACCGGCACGCACGGCACGCCGAACAATCATCACGGCACCACCGGCAAGGCCCGCAACCGCGGCTAA

>XooPXO524

TTGGCTGTTGCAAGCGCTGCTGCGGTGCGCCATCCTGCGCGCACTATCAGTGTGGGTGAACGTTCCATGGCCGGACTTCGCATCATCAAGAAGTATCCCAATCGCCGTCTCTACGACACGGAAATCTCCAGCTATATCACCATCGAAGATGTGCGCCAATTGATCATCGATGGCGAAGAATTCGAGGTACGCGATGCCAAGAGTGGCGAAGACCTGAGCCGCGCGGTGCTGCTGCAAATCATCGCCGACCGCGAACAGGACGGCGAGCCGATGCTCTCCACCCAGCTGCTGAGCCAGATCATCCGGTTCTATGGCGATTCGCTGCAGGGCTTCATGGGCAACTACCTGGAGCGCAGCATGCAGGTGTTCCTGGATCAGCAGCAGCAGTTCCGCCAGCAGATGGGCAACCTGCTCGGGCAGACGCCATGGGCGATGATGAACCAGCTGACCGAGCGCAACCTGGAGTTGTGGCAGGAGTTCCAGCGCAATTTCGGCGCCGGCTTCGGTCGCCCGGTTGGCCCTGGCACGCCGCCGAACCCGCCCGGTGCAAGCGGCCTGGGCAGCGGCCCGATTGGAACCGGCACGCACGGCACGCCGAACAATCATCACGGCACCACCGGCAAGGCCCGCAACCGCGGCTAA

>XooPXO282

TTGGCTGTTGCAAGCGCTGCTGCGGTGCGCCATCCTGCGCGCACTATCAGTGTGGGTGAACGTTCCATGGCCGGACTTCGCATCATCAAGAAGTATCCCAATCGCCGTCTCTACGACACGGAAATCTCCAGCTATATCACCATCGAAGATGTGCGCCAATTGATCATCGATGGCGAAGAATTCGAGGTACGCGATGCCAAGAGTGGCGAAGACCTGAGCCGCGCGGTGCTGCTGCAAATCATCGCCGACCGCGAACAGGACGGCGAGCCGATGCTCTCCACCCAGCTGCTGAGCCAGATCATCCGGTTCTATGGCGATTCGCTGCAGGGCTTCATGGGCAACTACCTGGAGCGCAGCATGCAGGTGTTCCTGGATCAGCAGCAGCAGTTCCGCCAGCAGATGGGCAACCTGCTCGGGCAGACGCCATGGGCGATGATGAACCAGCTGACCGAGCGCAACCTGGAGTTGTGGCAGGAGTTCCAGCGCAATTTCGGCGCCGGCTTCGGTCGCCCGGTTGGCCCTGGCACGCCGCCGAACCCGCCCGGTGCAAGCGGCCTGGGCAGCGGCCCGATTGGAACCGGCACGCACGGCACGCCGAACAATCATCACGGCACCACCGGCAAGGCCCGCAACCGCGGCTAA

>XooPXO236

TTGGCTGTTGCAAGCGCTGCTGCGGTGCGCCATCCTGCGCGCACTATCAGTGTGGGTGAACGTTCCATGGCCGGACTTCGCATCATCAAGAAGTATCCCAATCGCCGTCTCTACGACACGGAAATCTCCAGCTATATCACCATCGAAGATGTGCGCCAATTGATCATCGATGGCGAAGAATTCGAGGTACGCGATGCCAAGAGTGGCGAAGACCTGAGCCGCGCGGTGCTGCTGCAAATCATCGCCGACCGCGAACAGGACGGCGAGCCGATGCTCTCCACCCAGCTGCTGAGCCAGATCATCCGGTTCTATGGCGATTCGCTGCAGGGCTTCATGGGCAACTACCTGGAGCGCAGCATGCAGGTGTTCCTGGATCAGCAGCAGCAGTTCCGCCAGCAGATGGGCAACCTGCTCGGGCAGACGCCATGGGCGATGATGAACCAGCTGACCGAGCGCAACCTGGAGTTGTGGCAGGAGTTCCAGCGCAATTTCGGCGCCGGCTTCGGTCGCCCGGTTGGCCCTGGCACGCCGCCGAACCCGCCCGGTGCAAGCGGCCTGGGCAGCGGCCCGATTGGAACCGGCACGCACGGCACGCCGAACAATCATCACGGCACCACCGGCAAGGCCCGCAACCGCGGCTAA

>XooPXO211

TTGGCTGTTGCAAGCGCTGCTGCGGTGCGCCATCCTGCGCGCACTATCAGTGTGGGTGAACGTTCCATGGCCGGACTTCGCATCATCAAGAAGTATCCCAATCGCCGTCTCTACGACACGGAAATCTCCAGCTATATCACCATCGAAGATGTGCGCCAATTGATCATCGATGGCGAAGAATTCGAGGTACGCGATGCCAAGAGTGGCGAAGACCTGAGCCGCGCGGTGCTGCTGCAAATCATCGCCGACCGCGAACAGGACGGCGAGCCGATGCTCTCCACCCAGCTGCTGAGCCAGATCATCCGGTTCTATGGCGATTCGCTGCAGGGCTTCATGGGCAACTACCTGGAGCGCAGCATGCAGGTGTTCCTGGATCAGCAGCAGCAGTTCCGCCAGCAGATGGGCAACCTGCTCGGGCAGACGCCATGGGCGATGATGAACCAGCTGACCGAGCGCAACCTGGAGTTGTGGCAGGAGTTCCAGCGCAATTTCGGCGCCGGCTTCGGTCGCCCGGTTGGCCCTGGCACGCCGCCGAACCCGCCCGGTGCAAGCGGCCTGGGCAGCGGCCCGATTGGAACCGGCACGCACGGCACGCCGAACAATCATCACGGCACCACCGGCAAGGCCCGCAACCGCGGCT AA

>XooPXO145

TTGGCTGTTGCAAGCGCTGCTGCGGTGCGCCATCCTGCGCGCACTATCAGTGTGGGTGAACGTTCCATGGCCGGACTTCGCATCATCAAGAAGTATCCCAATCGCCGTCTCTACGACACGGAAATCTCCAGCTATATCACCATCGAAGATGTGCGCCAATTGATCATCGATGGCGAAGAATTCGAGGTACGCGATGCCAAGAGTGGCGAAGACCTGAGCCGCGCGGTGCTGCTGCAAATCATCGCCGACCGCGAACAGGACGGCGAGCCGATGCTCTCCACCCAGCTGCTGAGCCAGATCATCCGGTTCTATGGCGATTCGCTGCAGGGCTTCATGGGCAACTACCTGGAGCGCAGCATGCAGGTGTTCCTGGATCAGCAGCAGCAGTTCCGCCAGCAGATGGGCAACCTGCTCGGGCAGACGCCATGGGCGATGATGAACCAGCTGACCGAGCGCAACCTGGAGTTGTGGCAGGAGTTCCAGCGCAATTTCGGCGCCGGCTTCGGTCGCCCGGTTGGCCCTGGCACGCCGCCGAACCCGCCCGGTGCAAGCGGCCTGGGCAGCGGCCCGATTGGAACCGGCACGCACGGCACGCCGAACAATCATCACGGCACCACCGGCAAGGCCCGCAACCGCGGCTAA

>XooPXO71

TTGGCTGTTGCAAGCGCTGCTGCGGTGCGCCATCCTGCGCGCACTATCAGTGTGGGTGAACGTTCCATGGCCGGACTTCGCATCATCAAGAAGTATCCCAATCGCCGTCTCTACGACACGGAAATCTCCAGCTATATCACCATCGAAGATGTGCGCCAATTGATCATCGATGGCGAAGAATTCGAGGTACGCGATGCCAAGAGTGGCGAAGACCTGAGCCGCGCGGTGCTGCTGCAAATCATCGCCGACCGCGAACAGGACGGCGAGCCGATGCTCTCCACCCAGCTGCTGAGCCAGATCATCCGGTTCTATGGCGATTCGCTGCAGGGCTTCATGGGCAACTACCTGGAGCGCAGCATGCAGGTGTTCCTGGATCAGCAGCAGCAGTTCCGCCAGCAGATGGGCAACCTGCTCGGGCAGACGCCATGGGCGATGATGAACCAGCTGACCGAGCGCAACCTGGAGTTGTGGCAGGAGTTCCAGCGCAATTTCGGCGCCGGCTTCGGTCGCCCGGTTGGCCCTGGCACGCCGCCGAACCCGCCCGGTGCAAGCGGCCTGGGCAGCGGCCCGATTGGAACCGGCACGCACGGCACGCCGAACAATCATCACGGCACCACCGGCAAGGCCCGCAACCGCGGCTAA

>XooPXO83

TTGGCTGTTGCAAGCGCTGCTGCGGTGCGCCATCCTGCGCGCACTATCAGTGTGGGTGAACGTTCCATGGCCGGACTTCGCATCATCAAGAAGTATCCCAATCGCCGTCTCTACGACACGGAAATCTCCAGCTATATCACCATCGAAGATGTGCGCCAATTGATCATCGATGGCGAAGAATTCGAGGTACGCGATGCCAAGAGTGGCGAAGACCTGAGCCGCGCGGTGCTGCTGCAAATCATCGCCGACCGCGAACAGGACGGCGAGCCGATGCTCTCCACCCAGCTGCTGAGCCAGATCATCCGGTTCTATGGCGATTCGCTGCAGGGCTTCATGGGCAACTACCTGGAGCGCAGCATGCAGGTGTTCCTGGATCAGCAGCAGCAGTTCCGCCAGCAGATGGGCAACCTGCTCGGGCAGACGCCATGGGCGATGATGAACCAGCTGACCGAGCGCAACCTGGAGTTGTGGCAGGAGTTCCAGCGCAATTTCGGCGCCGGCTTCGGTCGCCCGGTTGGCCCTGGCACGCCGCCGAACCCGCCCGGTGCAAGCGGCCTGGGCAGCGGCCCGATTGGAACCGGCACGCACGGCACGCCGAACAATCATCACGGCACCACCGGCAAGGCCCGCAACCGCGGCTAA

>XooPXO86

TTGGCTGTTGCAAGCGCTGCTGCGGTGCGCCATCCTGCGCGCACTATCAGTGTGGGTGAACGTTCCATGGCCGGACTTCGCATCATCAAGAAGTATCCCAATCGCCGTCTCTACGACACGGAAATCTCCAGCTATATCACCATCGAAGATGTGCGCCAATTGATCATCGATGGCGAAGAATTCGAGGTACGCGATGCCAAGAGTGGCGAAGACCTGAGCCGCGCGGTGCTGCTGCAAATCATCGCCGACCGCGAACAGGACGGCGAGCCGATGCTCTCCACCCAGCTGCTGAGCCAGATCATCCGGTTCTATGGCGATTCGCTGCAGGGCTTCATGGGCAACTACCTGGAGCGCAGCATGCAGGTGTTCCTGGATCAGCAGCAGCAGTTCCGCCAGCAGATGGGCAACCTGCTCGGGCAGACGCCATGGGCGATGATGAACCAGCTGACCGAGCGCAACCTGGAGTTGTGGCAGGAGTTCCAGCGCAATTTCGGCGCCGGCTTCGGTCGCCCGGTTGGCCCTGGCACGCCGCCGAACCCGCCCGGTGCAAGCGGCCTGGGCAGCGGCCCGATTGGAACCGGCACGCACGGCACGCCGAACAATCATCACGGCACCACCGGCAAGGCCCGCAACCGCGGCTAA

>XooMAFF 311018

TTGGCTGTTGCAAGCGCTGCTGCGGTGCGCCATCCTGCGCGCACTATCAGTGTGGGTGAACGTTCCATGGCCGGACTTCGCATCATCAAGAAGTATCCCAATCGCCGTCTCTACGACACGGAAATCTCCAGCTATATCACCATCGAAGATGTGCGCCAATTGATCATCGATGGCGAAGAATTCGAGGTACGCGATGCCAAGAGTGGCGAAGACCTGAGCCGCGCGGTGCTGCTGCAAATCATCGCCGACCGCGAACAGGACGGCGAGCCGATGCTCTCCACCCAGCTGCTGAGCCAGATCATCCGGTTCTATGGCGATTCGCTGCAGGGCTTCATGGGCAACTACCTGGAGCGCAGCATGCAGGTGTTCCTGGATCAGCAGCAGCAGTTCCGCCAGCAGATGGGCAACCTGCTCGGGCAGACGCCATGGGCGATGATGAACCAGCTGACCGAGCGCAACCTGGAGTTGTGGCAGGAGTTCCAGCGCAATTTCGGCGCCGGCTTCGGTCGCCCGGTTGGCCCTGGCACGCCGCCGAACCCGCCCGGTGCAAGCGGCCTGGGCAGCGGCCCGATTGGAACCGGCACGCACGGCACGCCGAACAATCATCACGGCACCACCGGCAAGGCCCGCAACCGCGGCTAA

>XooKACC 10331

TTGGCTGTTGCAAGCGCTGCTGCGGTGCGCCATCCTGCGCGCACTATCAGTGTGGGTGAACGTTCCATGGCCGGACTTCGCATCATCAAGAAGTATCCCAATCGCCGTCTCTACGACACGGAAATCTCCAGCTATATCACCATCGAAGATGTGCGCCAATTGATCATCGATGGCGAAGAATTCGAGGTACGCGATGCCAAGAGTGGCGAAGACCTGAGCCGCGCGGTGCTGCTGCAAATCATCGCCGACCGCGAACAGGACGGCGAGCCGATGCTCTCCACCCAGCTGCTGAGCCAGATCATCCGGTTCTATGGCGATTCGCTGCAGGGCTTCATGGGCAACTACCTGGAGCGCAGCATGCAGGTGTTCCTGGATCAGCAGCAGCAGTTCCGCCAGCAGATGGGCAACCTGCTCGGGCAGACGCCATGGGCGATGATGAACCAGCTGACCGAGCGCAACCTGGAGTTGTGGCAGGAGTTCCAGCGCAATTTCGGCGCCGGCTTCGGTCGCCCGGTTGGCCCTGGCACGCCGCCGAACCCGCCCGGTGCAAGCGGCCTGGGCAGCGGCCCGATTGGAACCGGCACGCACGGCACGCCGAACAATCATCACGGCACCACCGGCAAGGCCCGCAACCGCGGCTAA

>XooMAI145

TTGGCTGTTGCAAGCGCTGCTGCGGTGCGCCATCCTGCGCGCACTATCAGTGTGGGTGAACGTTCCATGGCCGGACTTCGCATCATCAAGAAGTACCCCAATCGCCGTCTCTACGACACGGAAATCTCCAGCTACATCACCATCGAAGACGTGCGCCAATTGATCATCGATGGCGAAGAATTCGAGGTACGCGACGCCAAGAGTGGCGAAGACCTGAGCCGCGCGGTGCTGCTGCAAATCATCGCCGACCGCGAACAGGACGGCGAGCCGATGCTCTCCACCCAGCTGCTGAGCCAGATCATCCGGTTCTATGGCGATTCGCTGCAGGGCTTCATGGGCAACTACCTGGAGCGCAGCATGCAGGTGTTCCTGGATCAGCAGCAGCAGTTCCGCCAGCAGATGGGCAACCTGCTCGGGCAGACGCCATGGGCGATGATGAACCAGCTGACAGAGCGCAACCTGGAGTTGTGGCAGGAGTTCCAGCGCAATTTCGGCACCGGCTTCGGTCGCCCGGGTGGCCCTGGCACGCCGCCGAACCCGCCCGGTGCAAGCGGCCTGGGCAGCGGCCCGATTGGAACCGGCACCCACGGCACGCCGAACAATAATCACGGCACCACCGGCAAGGCCCGCAACCGCGGCTAA

>XooMAI134

TTGGCTGTTGCAAGCGCTGCTGCGGTGCGCCATCCTGCGCGCACTATCAGTGTGGGTGAACGTTCCATGGCCGGACTTCGCATCATCAAGAAGTACCCCAATCGCCGTCTCTACGACACGGAAATCTCCAGCTACATCACCATCGAAGACGTGCGCCAATTGATCATCGATGGCGAAGAATTCGAGGTACGCGACGCCAAGAGTGGCGAAGACCTGAGCCGCGCGGTGCTGCTGCAAATCATCGCCGACCGCGAACAGGACGGCGAGCCGATGCTCTCCACCCAGCTGCTGAGCCAGATCATCCGGTTCTATGGCGATTCGCTGCAGGGCTTCATGGGCAACTACCTGGAGCGCAGCATGCAGGTGTTCCTGGATCAGCAGCAGCAGTTCCGCCAGCAGATGGGCAACCTGCTCGGGCAGACGCCATGGGCGATGATGAACCAGCTGACAGAGCGCAACCTGGAGTTGTGGCAGGAGTTCCAGCGCAATTTCGGCACCGGCTTCGGTCGCCCGGGTGGCCCTGGCACGCCGCCGAACCCGCCCGGTGCAAGCGGCCTGGGCAGCGGCCCGATTGGAACCGGCACCCACGGCACGCCGAACAATAATCACGGCACCACCGGCAAGGCCCGCAACCGCGGCTAA

>XooMAI129

TTGGCTGTTGCAAGCGCTGCTGCGGTGCGCCATCCTGCGCGCACTATCAGTGTGGGTGAACGTTCCATGGCCGGACTTCGCATCATCAAGAAGTACCCCAATCGCCGTCTCTACGACACGGAAATCTCCAGCTACATCACCATCGAAGACGTGCGCCAATTGATCATCGATGGCGAAGAATTCGAGGTACGCGACGCCAAGAGTGGCGAAGACCTGAGCCGCGCGGTGCTGCTGCAAATCATCGCCGACCGCGAACAGGACGGCGAGCCGATGCTCTCCACCCAGCTGCTGAGCCAGATCATCCGGTTCTATGGCGATTCGCTGCAGGGCTTCATGGGCAACTACCTGGAGCGCAGCATGCAGGTGTTCCTGGATCAGCAGCAGCAGTTCCGCCAGCAGATGGGCAACCTGCTCGGGCAGACGCCATGGGCGATGATGAACCAGCTGACAGAGCGCAACCTGGAGTTGTGGCAGGAGTTCCAGCGCAATTTCGGCACCGGCTTCGGTCGCCCGGGTGGCCCTGGCACGCCGCCGAACCCGCCCGGTGCAAGCGGCCTGGGCAGCGGCCCGATTGGAACCGGCACCCACGGCACGCCGAACAATAATCACGGCACCACCGGCAAGGCCCGCAACCGCGGCTAA

>XooMAI106

TTGGCTGTTGCAAGCGCTGCTGCGGTGCGCCATCCTGCGCGCACTATCAGTGTGGGTGAACGTTCCATGGCCGGACTTCGCATCATCAAGAAGTACCCCAATCGCCGTCTCTACGACACGGAAATCTCCAGCTACATCACCATCGAAGACGTGCGCCAATTGATCATCGATGGCGAAGAATTCGAGGTACGCGACGCCAAGAGTGGCGAAGACCTGAGCCGCGCGGTGCTGCTGCAAATCATCGCCGACCGCGAACAGGACGGCGAGCCGATGCTCTCCACCCAGCTGCTGAGCCAGATCATCCGGTTCTATGGCGATTCGCTGCAGGGCTTCATGGGCAACTACCTGGAGCGCAGCATGCAGGTGTTCCTGGATCAGCAGCAGCAGTTCCGCCAGCAGATGGGCAACCTGCTCGGGCAGACGCCATGGGCGATGATGAACCAGCTGACAGAGCGCAACCTGGAGTTGTGGCAGGAGTTCCAGCGCAATTTCGGCACCGGCTTCGGTCGCCCGGGTGGCCCTGGCACGCCGCCGAACCCGCCCGGTGCAAGCGGCCTGGGCAGCGGCCCGATTGGAACCGGCACCCACGGCACGCCGAACAATAATCACGGCACCACCGGCAAGGCCCGCAACCGCGGCTAA

>XooMAI99

TTGGCTGTTGCAAGCGCTGCTGCGGTGCGCCATCCTGCGCGCACTATCAGTGTGGGTGAACGTTCCATGGCCGGACTTCGCATCATCAAGAAGTACCCCAATCGCCGTCTCTACGACACGGAAATCTCCAGCTACATCACCATCGAAGACGTGCGCCAATTGATCATCGATGGCGAAGAATTCGAGGTACGCGACGCCAAGAGTGGCGAAGACCTGAGCCGCGCGGTGCTGCTGCAAATCATCGCCGACCGCGAACAGGACGGCGAGCCGATGCTCTCCACCCAGCTGCTGAGCCAGATCATCCGGTTCTATGGCGATTCGCTGCAGGGCTTCATGGGCAACTACCTGGAGCGCAGCATGCAGGTGTTCCTGGATCAGCAGCAGCAGTTCCGCCAGCAGATGGGCAACCTGCTCGGGCAGACGCCATGGGCGATGATGAACCAGCTGACAGAGCGCAACCTGGAGTTGTGGCAGGAGTTCCAGCGCAATTTCGGCACCGGCTTCGGTCGCCCGGGTGGCCCTGGCACGCCGCCGAACCCGCCCGGTGCAAGCGGCCTGGGCAGCGGCCCGATTGGAACCGGCACCCACGGCACGCCGAACAATAATCACGGCACCACCGGCAAGGCCCGCAACCGCGGCTAA

>XooMAI95

TTGGCTGTTGCAAGCGCTGCTGCGGTGCGCCATCCTGCGCGCACTATCAGTGTGGGTGAACGTTCCATGGCCGGACTTCGCATCATCAAGAAGTACCCCAATCGCCGTCTCTACGACACGGAAATCTCCAGCTACATCACCATCGAAGACGTGCGCCAATTGATCATCGATGGCGAAGAATTCGAGGTACGCGACGCCAAGAGTGGCGAAGACCTGAGCCGCGCGGTGCTGCTGCAAATCATCGCCGACCGCGAACAGGACGGCGAGCCGATGCTCTCCACCCAGCTGCTGAGCCAGATCATCCGGTTCTATGGCGATTCGCTGCAGGGCTTCATGGGCAACTACCTGGAGCGCAGCATGCAGGTGTTCCTGGATCAGCAGCAGCAGTTCCGCCAGCAGATGGGCAACCTGCTCGGGCAGACGCCATGGGCGATGATGAACCAGCTGACAGAGCGCAACCTGGAGTTGTGGCAGGAGTTCCAGCGCAATTTCGGCACCGGCTTCGGTCGCCCGGGTGGCCCTGGCACGCCGCCGAACCCGCCCGGTGCAAGCGGCCTGGGCAGCGGCCCGATTGGAACCGGCACCCACGGCACGCCGAACAATAATCACGGCACCACCGGCAAGGCCCGCAACCGCGGCTAA

>XooMAI73

TTGGCTGTTGCAAGCGCTGCTGCGGTGCGCCATCCTGCGCGCACTATCAGTGTGGGTGAACGTTCCATGGCCGGACTTCGCATCATCAAGAAGTACCCCAATCGCCGTCTCTACGACACGGAAATCTCCAGCTACATCACCATCGAAGACGTGCGCCAATTGATCATCGATGGCGAAGAATTCGAGGTACGCGACGCCAAGAGTGGCGAAGACCTGAGCCGCGCGGTGCTGCTGCAAATCATCGCCGACCGCGAACAGGACGGCGAGCCGATGCTCTCCACCCAGCTGCTGAGCCAGATCATCCGGTTCTATGGCGATTCGCTGCAGGGCTTCATGGGCAACTACCTGGAGCGCAGCATGCAGGTGTTCCTGGATCAGCAGCAGCAGTTCCGCCAGCAGATGGGCAACCTGCTCGGGCAGACGCCATGGGCGATGATGAACCAGCTGACAGAGCGCAACCTGGAGTTGTGGCAGGAGTTCCAGCGCAATTTCGGCACCGGCTTCGGTCGCCCGGGTGGCCCTGGCACGCCGCCGAACCCGCCCGGTGCAAGCGGCCTGGGCAGCGGCCCGATTGGAACCGGCACCCACGGCACGCCGAACAATAATCACGGCACCACCGGCAAGGCCCGCAACCGCGGCTAA

>XooMAI68

TTGGCTGTTGCAAGCGCTGCTGCGGTGCGCCATCCTGCGCGCACTATCAGTGTGGGTGAACGTTCCATGGCCGGACTTCGCATCATCAAGAAGTACCCCAATCGCCGTCTCTACGACACGGAAATCTCCAGCTACATCACCATCGAAGACGTGCGCCAATTGATCATCGATGGCGAAGAATTCGAGGTACGCGACGCCAAGAGTGGCGAAGACCTGAGCCGCGCGGTGCTGCTGCAAATCATCGCCGACCGCGAACAGGACGGCGAGCCGATGCTCTCCACCCAGCTGCTGAGCCAGATCATCCGGTTCTATGGCGATTCGCTGCAGGGCTTCATGGGCAACTACCTGGAGCGCAGCATGCAGGTGTTCCTGGATCAGCAGCAGCAGTTCCGCCAGCAGATGGGCAACCTGCTCGGGCAGACGCCATGGGCGATGATGAACCAGCTGACAGAGCGCAACCTGGAGTTGTGGCAGGAGTTCCAGCGCAATTTCGGCACCGGCTTCGGTCGCCCGGGTGGCCCTGGCACGCCGCCGAACCCGCCCGGTGCAAGCGGCCTGGGCAGCGGCCCGATTGGAACCGGCACCCACGGCACGCCGAACAATAATCACGGCACCACCGGCAAGGCCCGCAACCGCGGCTAA

>XooAXO1947

TTGGCTGTTGCAAGCGCTGCTGCGGTGCGCCATCCTGCGCGCACTATCAGTGTGGGTGAACGTTCCATGGCCGGACTTCGCATCATCAAGAAGTACCCCAATCGCCGTCTCTACGACACGGAAATCTCCAGCTACATCACCATCGAAGACGTGCGCCAATTGATCATCGATGGCGAAGAATTCGAGGTACGCGACGCCAAGAGTGGCGAAGACCTGAGCCGCGCGGTGCTGCTGCAAATCATCGCCGACCGCGAACAGGACGGCGAGCCGATGCTCTCCACCCAGCTGCTGAGCCAGATCATCCGGTTCTATGGCGATTCGCTGCAGGGCTTCATGGGCAACTACCTGGAGCGCAGCATGCAGGTGTTCCTGGATCAGCAGCAGCAGTTCCGCCAGCAGATGGGCAACCTGCTCGGGCAGACGCCATGGGCGATGATGAACCAGCTGACAGAGCGCAACCTGGAGTTGTGGCAGGAGTTCCAGCGCAATTTCGGCACCGGCTTCGGTCGCCCGGGTGGCCCTGGCACGCCGCCGAACCCGCCCGGTGCAAGCGGCCTGGGCAGCGGCCCGATTGGAACCGGCACCCACGGCACGCCGAACAATAATCACGGCACCACCGGCAAGGCCCGCAACCGCGGCTAA

>XocCFBP2286

TTGGCTGTTGCAAGCGCTGCTGCGGTGCGCCATCCTGCGCGCACTATCAGTGTGGGTGAACGTTCCATGGCCGGACTTCGCATCATCAAGAAGTATCCCAATCGCCGTCTCTACGACACGGAAATCTCCAGCTACATCACCATCGAAGATGTGCGCCAATTGATCATCGATGGCGAAGAATTCGAGGTACGCGACGCCAAGAGTGGCGAAGACCTGAGCCGCGCGGTGCTGCTGCAAATCATCGCCGACCGCGAACAGGACGGCGAGCCGATGCTCTCCACCCAGCTGCTGAGCCAGATCATCCGGTTCTATGGCGATTCGCTGCAGGGCTTCATGGGTAACTACCTGGAGCGCAGCATGCAGGTGTTCCTGGATCAGCAGCAGCAGTTCCGCCAGCAGATGGGCAACCTGCTCGGGCAGACGCCATGGGCGATGATGAACCAGCTGACCGAGCGCAACCTGGAGTTGTGGCAGGAGTTCCAGCGCAATTTCGGCGCCGGCTTCGGTCGCCCGGTTGGCCCTGGCACGCCGCCGAACCCGCCCGGTGCAAGCAGCCTGGGCAGCGGCCCGATTGGAACCGGCACCCACGGCACGCCGAACAATCATCACGGCACCACCGGCAAGGCCCGCAACCGCGGCTAA

>XocRS105

TTGGCTGTTGCAAGCGCTGCTGCGGTGCGCCATCCTGCGCGCACTATCAGTGTGGGTGAACGTTCCATGGCCGGACTTCGCATCATCAAGAAGTATCCCAATCGCCGTCTCTACGACACGGAAATCTCCAGCTACATCACCATCGAAGATGTGCGCCAATTGATCATCGATGGCGAAGAATTCGAGGTACGCGACGCCAAGAGTGGCGAAGACCTGAGCCGCGCGGTGCTGCTGCAAATCATCGCCGACCGCGAACAGGACGGCGAGCCGATGCTCTCCACCCAGCTGCTGAGCCAGATCATCCGGTTCTATGGCGATTCGCTGCAGGGCTTCATGGGTAACTACCTGGAGCGCAGCATGCAGGTGTTCCTGGATCAGCAGCAGCAGTTCCGCCAGCAGATGGGCAACCTGCTCGGGCAGACGCCATGGGCGATGATGAACCAGCTGACCGAGCGCAACCTGGAGTTGTGGCAGGAGTTCCAGCGCAATTTCGGCGCCGGCTTCGGTCGCCCGGTTGGCCCTGGCACGCCGCCGAACCCGCCCGGTGCAAGCAGCCTGGGCAGCGGCCCGATTGGAACCGGCACCCACGGCACGCCGAACAATCATCACGGCACCACCGGCAAGGCCCGCAACCGCGGCTAA

>XocL8

TTGGCTGTTGCAAGCGCTGCTGCGGTGCGCCATCCTGCGCGCACTATCAGTGTGGGTGAACGTTCCATGGCCGGACTTCGCATCATCAAGAAGTATCCCAATCGCCGTCTCTACGACACGGAAATCTCCAGCTACATCACCATCGAAGATGTGCGCCAATTGATCATCGATGGCGAAGAATTCGAGGTACGCGACGCCAAGAGTGGCGAAGACCTGAGCCGCGCGGTGCTGCTGCAAATCATCGCCGACCGCGAACAGGACGGCGAGCCGATGCTCTCCACCCAGCTGCTGAGCCAGATCATCCGGTTCTATGGCGATTCGCTGCAGGGCTTCATGGGTAACTACCTGGAGCGCAGCATGCAGGTGTTCCTGGATCAGCAGCAGCAGTTCCGCCAGCAGATGGGCAACCTGCTCGGGCAGACGCCATGGGCGATGATGAACCAGCTGACCGAGCGCAACCTGGAGTTGTGGCAGGAGTTCCAGCGCAATTTCGGCGCCGGCTTCGGTCGCCCGGTTGGCCCTGGCACGCCGCCGAACCCGCCCGGTGCAAGCAGCCTGGGCAGCGGCCCGATTGGAACCGGCACCCACGGCACGCCGAACAATCATCACGGCACCACCGGCAAGGCCCGCAACCGCGGCTAA

>XocCFBP7341

TTGGCTGTTGCAAGCGCTGCTGCGGTGCGCCATCCTGCGCGCACTATCAGTGTGGGTGAACGTTCCATGGCCGGACTTCGCATCATCAAGAAGTATCCCAATCGCCGTCTCTACGACACGGAAATCTCCAGCTACATCACCATCGAAGATGTGCGCCAATTGATCATCGATGGCGAAGAATTCGAGGTACGCGACGCCAAGAGTGGCGAAGACCTGAGCCGCGCGGTGCTGCTGCAAATCATCGCCGACCGCGAACAGGACGGCGAGCCGATGCTCTCCACCCAGCTGCTGAGCCAGATCATCCGGTTCTATGGCGATTCGCTGCAGGGCTTCATGGGTAACTACCTGGAGCGCAGCATGCAGGTGTTCCTGGATCAGCAGCAGCAGTTCCGCCAGCAGATGGGCAACCTGCTCGGGCAGACGCCATGGGCGATGATGAACCAGCTGACCGAGCGCAACCTGGAGTTGTGGCAGGAGTTCCAGCGCAATTTCGGCGCCGGCTTCGGTCGCCCGGTTGGCCCTGGCACGCCGCCGAACCCGCCCGGTGCAAGCAGCCTGGGCAGCGGCCCGATTGGAACCGGCACCCACGGCACGCCGAACAATCATCACGGCACCACCGGCAAGGCCCGCAACCGCGGCTAA

>XocCFBP7331

TTGGCTGTTGCAAGCGCTGCTGCGGTGCGCCATCCTGCGCGCACTATCAGTGTGGGTGAACGTTCCATGGCCGGACTTCGCATCATCAAGAAGTATCCCAATCGCCGTCTCTACGACACGGAAATCTCCAGCTACATCACCATCGAAGATGTGCGCCAATTGATCATCGATGGCGAAGAATTCGAGGTACGCGACGCCAAGAGTGGCGAAGACCTGAGCCGCGCGGTGCTGCTGCAAATCATCGCCGACCGCGAACAGGACGGCGAGCCGATGCTCTCCACCCAGCTGCTGAGCCAGATCATCCGGTTCTATGGCGATTCGCTGCAGGGCTTCATGGGTAACTACCTGGAGCGCAGCATGCAGGTGTTCCTGGATCAGCAGCAGCAGTTCCGCCAGCAGATGGGCAACCTGCTCGGGCAGACGCCATGGGCGATGATGAACCAGCTGACCGAGCGCAACCTGGAGTTGTGGCAGGAGTTCCAGCGCAATTTCGGCGCCGGCTTCGGTCGCCCGGTTGGCCCTGGCACGCCGCCGAACCCGCCCGGTGCAAGCAGCCTGGGCAGCGGCCCGATTGGAACCGGCACCCACGGCACGCCGAACAATCATCACGGCACCACCGGCAAGGCCCGCAACCGCGGCTAA

>XocBXOR1

TTGGCTGTTGCAAGCGCTGCTGCGGTGCGCCATCCTGCGCGCACTATCAGTGTGGGTGAACGTTCCATGGCCGGACTTCGCATCATCAAGAAGTATCCCAATCGCCGTCTCTACGACACGGAAATCTCCAGCTACATCACCATCGAAGATGTGCGCCAATTGATCATCGATGGCGAAGAATTCGAGGTACGCGACGCCAAGAGTGGCGAAGACCTGAGCCGCGCGGTGCTGCTGCAAATCATCGCCGACCGCGAACAGGACGGCGAGCCGATGCTCTCCACCCAGCTGCTGAGCCAGATCATCCGGTTCTATGGCGATTCGCTGCAGGGCTTCATGGGTAACTACCTGGAGCGCAGCATGCAGGTGTTCCTGGATCAGCAGCAGCAGTTCCGCCAGCAGATGGGCAACCTGCTCGGGCAGACGCCATGGGCGATGATGAACCAGCTGACCGAGCGCAACCTGGAGTTGTGGCAGGAGTTCCAGCGCAATTTCGGCGCCGGCTTCGGTCGCCCGGTTGGCCCTGGCACGCCGCCGAACCCGCCCGGTGCAAGCAGCCTGGGCAGCGGCCCGATTGGAACCGGCACCCACGGCACGCCGAACAATCATCACGGCACCACCGGCAAGGCCCGCAACCGCGGCTAA

>XocBLS279

TTGGCTGTTGCAAGCGCTGCTGCGGTGCGCCATCCTGCGCGCACTATCAGTGTGGGTGAACGTTCCATGGCCGGACTTCGCATCATCAAGAAGTATCCCAATCGCCGTCTCTACGACACGGAAATCTCCAGCTACATCACCATCGAAGATGTGCGCCAATTGATCATCGATGGCGAAGAATTCGAGGTACGCGACGCCAAGAGTGGCGAAGACCTGAGCCGCGCGGTGCTGCTGCAAATCATCGCCGACCGCGAACAGGACGGCGAGCCGATGCTCTCCACCCAGCTGCTGAGCCAGATCATCCGGTTCTATGGCGATTCGCTGCAGGGCTTCATGGGTAACTACCTGGAGCGCAGCATGCAGGTGTTCCTGGATCAGCAGCAGCAGTTCCGCCAGCAGATGGGCAACCTGCTCGGGCAGACGCCATGGGCGATGATGAACCAGCTGACCGAGCGCAACCTGGAGTTGTGGCAGGAGTTCCAGCGCAATTTCGGCGCCGGCTTCGGTCGCCCGGTTGGCCCTGGCACGCCGCCGAACCCGCCCGGTGCAAGCAGCCTGGGCAGCGGCCCGATTGGAACCGGCACCCACGGCACGCCGAACAATCATCACGGCACCACCGGCAAGGCCCGCAACCGCGGCTAA

>XocB8-12

TTGGCTGTTGCAAGCGCTGCTGCGGTGCGCCATCCTGCGCGCACTATCAGTGTGGGTGAACGTTCCATGGCCGGACTTCGCATCATCAAGAAGTATCCCAATCGCCGTCTCTACGACACGGAAATCTCCAGCTACATCACCATCGAAGATGTGCGCCAATTGATCATCGATGGCGAAGAATTCGAGGTACGCGACGCCAAGAGTGGCGAAGACCTGAGCCGCGCGGTGCTGCTGCAAATCATCGCCGACCGCGAACAGGACGGCGAGCCGATGCTCTCCACCCAGCTGCTGAGCCAGATCATCCGGTTCTATGGCGATTCGCTGCAGGGCTTCATGGGTAACTACCTGGAGCGCAGCATGCAGGTGTTCCTGGATCAGCAGCAGCAGTTCCGCCAGCAGATGGGCAACCTGCTCGGGCAGACGCCATGGGCGATGATGAACCAGCTGACCGAGCGCAACCTGGAGTTGTGGCAGGAGTTCCAGCGCAATTTCGGCGCCGGCTTCGGTCGCCCGGTTGGCCCTGGCACGCCGCCGAACCCGCCCGGTGCAAGCAGCCTGGGCAGCGGCCCGATTGGAACCGGCACCCACGGCACGCCGAACAATCATCACGGCACCACCGGCAAGGCCCGCAACCGCGGCTAA

>XocBLS256

TTGGCTGTTGCAAGCGCTGCTGCGGTGCGCCATCCTGCGCGCACTATCAGTGTGGGTGAACGTTCCATGGCCGGACTTCGCATCATCAAGAAGTATCCCAATCGCCGTCTCTACGACACGGAAATCTCCAGCTACATCACCATCGAAGATGTGCGCCAATTGATCATCGATGGCGAAGAATTCGAGGTACGCGACGCCAAGAGTGGCGAAGACCTGAGCCGCGCGGTGCTGCTGCAAATCATCGCCGACCGCGAACAGGACGGCGAGCCGATGCTCTCCACCCAGCTGCTGAGCCAGATCATCCGGTTCTATGGCGATTCGCTGCAGGGCTTCATGGGTAACTACCTGGAGCGCAGCATGCAGGTGTTCCTGGATCAGCAGCAGCAGTTCCGCCAGCAGATGGGCAACCTGCTCGGGCAGACGCCATGGGCGATGATGAACCAGCTGACCGAGCGCAACCTGGAGTTGTGGCAGGAGTTCCAGCGCAATTTCGGCGCCGGCTTCGGTCGCCCGGTTGGCCCTGGCACGCCGCCGAACCCGCCCGGTGCAAGCAGCCTGGGCAGCGGCCCGATTGGAACCGGCACCCACGGCACGCCGAACAATCATCACGGCACCACCGGCAAGGCCCGCAACCGCGGCTAA

>XocYM15

TTGGCTGTTGCAAGCGCTGCTGCGGTGCGCCATCCTGCGCGCACTATCAGTGTGGGTGAACGTTCCATGGCCGGACTTCGCATCATCAAGAAGTATCCCAATCGCCGTCTCTACGACACGGAAATCTCCAGCTACATCACCATCGAAGATGTGCGCCAATTGATCATCGATGGCGAAGAATTCGAGGTACGCGACGCCAAGAGTGGCGAAGACCTGAGCCGCGCGGTGCTGCTGCAAATCATCGCCGACCGCGAACAGGACGGCGAGCCGATGCTCTCCACCCAGCTGCTGAGCCAGATCATCCGGTTCTATGGCGATTCGCTGCAGGGCTTCATGGGTAACTACCTGGAGCGCAGCATGCAGGTGTTCCTGGATCAGCAGCAGCAGTTCCGCCAGCAGATGGGCAACCTGCTCGGGCAGACGCCATGGGCGATGATGAACCAGCTGACCGAGCGCAACCTGGAGTTGTGGCAGGAGTTCCAGCGCAATTTCGGCGCCGGCTTCGGTCGCCCGGTTGGCCCTGGCACGCCGCCGAACCCGCCCGGTGCAAGCAGCCTGGGCAGCGGCCCGATTGGAACCGGCACCCACGGCACGCCGAACAATCATCACGGCACCACCGGCAAGGCCCGCAACCGCGGCTAA

>XocCFBP7342

TTGGCTGTTGCAAGCGCTGCTGCGGTGCGCCATCCTGCGCGCACTATCAGTGTGGGTGAACGTTCCATGGCCGGACTTCGCATCATCAAGAAGTATCCCAATCGCCGTCTCTACGACACGGAAATCTCCAGCTACATCACCATCGAAGATGTGCGCCAATTGATCATCGATGGCGAAGAATTCGAGGTACGCGACGCCAAGAGTGGCGAAGACCTGAGCCGCGCGGTGCTGCTGCAAATCATCGCCGACCGCGAACAGGACGGCGAGCCGATGCTCTCCACCCAGCTGCTGAGCCAGATCATCCGGTTCTATGGCGATTCGCTGCAGGGCTTCATGGGTAACTACCTGGAGCGCAGCATGCAGGTGTTCCTGGATCAGCAGCAGCAGTTCCGCCAGCAGATGGGCAACCTGCTCGGGCAGACGCCATGGGCGATGATGAACCAGCTGACCGAGCGCAACCTGGAGTTGTGGCAGGAGTTCCAGCGCAATTTCGGCGCCGGCTTCGGTCGCCCGGTTGGCCCTGGCACGCCGCCGAACCCGCCCGGTGCAAGCAGCCTGGGCAGCGGCCCGATTGGAACCGGCACCCACGGCACGCCGAACAATCATCACGGCACCACCGGCAAGGCCCGCAACCGCGGCTAA

>XppCFBP6982

TTGGCTGTTGCAAGCGCTGCTGCGGTGCGCCATCCTGCGCGCACTATCAGTGTGGGTGAACGTTCCATGGCCGGACTTCGCATCATCAAGAAGTATCCCAATCGCCGTCTCTACGACACGGAAATCTCCAGCTACATCACCATCGAAGATGTGCGCCAATTGATCATCGATGGCGAAGAATTCGAAGTACGCGACGCCAAGAGTGGCGAAGACCTCAGCCGTGCAGTGCTGCTGCAGATCATCGCCGACCGCGAACAGGACGGCGAGCCGATGCTCTCCACCCAGTTGCTGAGCCAGATCATCCGGTTTTATGGCGACTCGCTGCAGGGCTTCATGGGCAACTACCTGGAGCGCAGCATGCAGGTGTTCCTGGACCAGCAGCAGCAGTTCCGTCAACAGATGGGCAACCTGCTCGGGCAGACCCCGTGGGCGATGATGAATCAGCTGACCGAGCGCAACCTGGAGTTGTGGCAGGAGTTCCAGCGCAACTTCGGCGCCGGCTTCGGTCGCCCGGGTGGCCCTGGCACGCCGCCGAACCCACCCGGGGCGAGCGGCCTGGGCAGTGGCCCGATGGGAACCGGCACGCACGGCTCGGCCGGTGGCAACCACGGCACGACCGGCAAGGCCCGCAACCGCGGCTAA

>Xp91-118

TTGGCTGTTGCAAGCGCTGCTGCGGTGCGCCATCCTGCGCGCACTATCAGTGTGGGTGAACGTTCCATGGCCGGACTTCGCATCATCAAGAAGTATCCCAATCGCCGTCTCTACGACACGGAAATCTCCAGCTACATCACCATCGAAGATGTGCGCCAATTGATCATCGATGGCGAAGAATTCGAAGTACGCGATGCCAAGAGCGGCGAGGACCTCAGCCGCGCAGTGCTGCTGCAAATCATCGCCGACCGCGAGCAGGACGGCGAGCCGATGCTCTCCACCCAGTTGCTGAGCCAGATCATCCGGTTTTATGGCGACTCGCTGCAGGGCTTCATGGGCAATTACCTGGAGCGCAGCATGCAGGTGTTCCTGGATCAGCAGCAGCAGTTCCGTCAGCAGATGGGCAACCTGCTCGGGCAGACCCCGTGGGCGATGATGAACCAGTTGACCGAGCGCAACCTGGAGTTGTGGCAGGAGTTCCAGCGCAACTTCGGCGCCGGCTTCGGCCGCCCGGGTGGCCCGGGCACACCGCCGAGCCCGCCTGGGGCGAGCGGCCTGGGCAGCGGCCCGATGGGAACCGGCACGCATGGCTCGGCTGGCGGCAATCACGGCACGACCGGCAAGGCCCGCAACCGCGGCTAA

>XadLMG695

TTGGCTGTTGCAAGCGCTGCTGCGGTGCGCCATCCTGCGCGCACTATCAGTGTGGGTGAACGTTCCATGGCCGGACTTCGCATCATCAAGAAGTATCCCAATCGCCGTCTCTACGACACGGAAATCTCCAGCTACATCACCATCGAAGATGTGCGCCAATTGATCATCGATGGCGAAGAATTCGAAGTACGCGACGCCAAAAGTGGCGAAGACCTCAGCCGTGCAGTGCTGCTGCAGATCATCGCCGACCGCGAGCAGGACGGCGAGCCGATGCTCTCCACCCAGTTGCTGAGCCAGATCATCCGGTTTTATGGCGACTCGCTGCAGGGCTTCATGGGCAACTACCTGGAGCGCAGCATGCAGGTGTTCCTCGACCAGCAGCAGCAGTTCCGTCAACAGATGGGCAACCTGCTCGGGCAGACCCCGTGGGCGATGATGAATCAGCTGACCGAGCGCAACCTGGAGTTGTGGCAGGAGTTCCAGCGCAACTTCGGCGCCGGCTTCGGTCGCCCGGGTGGCCCTGGCACGCCGCCGAACCCACCCGGGGCGAGCGGCCTGGGCAGTGGCCCGATGGGAACCGGCACGCACGGCTCGGCCGGTGGCAACCACGGCACGACCGGCAAGGCCCGCAACCGCGGCTAA

>XeLMG930

TTGGCTGTTGCAAGCGCTGCTGCGGTGCGCCATCCTGCGCGCACTATCAGTGTGGGTGAACGTTCCATGGCCGGACTTCGCATCATCAAGAAGTATCCCAATCGCCGTCTCTACGACACGGAAATCTCCAGCTACATCACCATCGAAGATGTGCGCCAATTGATCATCGATGGCGAAGAATTCGAAGTACGCGATGCCAAGAGCGGCGAGGACCTCAGCCGCGCAGTGCTGCTGCAAATCATCGCCGACCGCGAGCAGGACGGCGAGCCGATGCTCTCCACCCAGTTGCTGAGCCAGATCATCCGGTTTTATGGCGACTCGCTGCAGGGCTTCATGGGCAATTACCTGGAGCGCAGCATGCAGGTGTTCCTGGATCAGCAGCAGCAGTTCCGTCAGCAGATGGGCAACCTGCTCGGGCAGACCCCGTGGGCGATGATGAACCAGTTGACCGAGCGCAACCTGGAGTTGTGGCAGGAGTTCCAGCGCAACTTCGGCGCCGGCTTCGGCCGCGCGGGTGGCCCGGGCACACCGCCGAGCCCGCCTGGGGCGAGCGGCCTGGGCAGCGGCCCGATGGGAACCGGCACGCATGGCTCGGCTGGCGGCAATCACGGCACGACCGGCAAGGCCCGCAACCGCGGCTAA

> Xcv85-10

TTGGCTGTTGCAAGCGCTGCTGCGGTGCGCCATCCTGCGCGCACTATCAGTGTGGGTGAACGTTCCATGGCCGGACTTCGCATCATCAAGAAGTATCCCAATCGCCGTCTCTACGACACGGAAATCTCCAGCTACATCACCATCGAAGATGTGCGCCAATTGATCATCGATGGCGAAGAATTCGAAGTACGCGATGCCAAGAGCGGCGAGGACCTCAGCCGCGCAGTGCTGCTGCAAATCATCGCCGACCGCGAGCAGGACGGCGAGCCGATGCTCTCCACCCAGTTGCTGAGCCAGATCATCCGGTTTTATGGCGACTCGCTGCAGGGCTTCATGGGCAATTACCTGGAGCGCAGCATGCAGGTGTTCCTGGATCAGCAGCAGCAGTTCCGTCAGCAGATGGGCAACCTGCTCGGGCAGACCCCGTGGGCGATGATGAACCAGTTGACCGAGCGCAACCTGGAGTTGTGGCAGGAGTTCCAGCGCAACTTCGGCGCCGGCTTCGGCCGCGCGGGTGGCCCGGGCACACCGCCGAGCCCGCCTGGGGCGAGCGGCCTGGGCAGCGGCCCGATGGGAACCGGCACGCATGGCTCGGCTGGCGGCAATCACGGCACGACCGGCAAGGCCCGCAACCGCGGCTAA

>XacF1

TTGGCTGTTGCAAGCGCTGCTGCGGTGCGCCATCCTGCGCGCACTATCAGTGTGGGTGAACGTTCCATGGCCGGACTTCGCATCATCAAGAAGTATCCCAATCGCCGTCTCTACGACACGGAAATCTCCAGCTACATCACCATCGAAGATGTGCGCCAATTGATCATCGATGGCGAAGAATTCGAAGTACGCGATGCCAAGAGCGGCGAGGACCTCAGCCGCGCAGTACTGCTGCAGATCATCGCCGACCGCGAGCAGGACGGCGAGCCGATGCTCTCCACCCAGTTGCTGAGCCAGATCATCCGGTTTTATGGCGACTCGCTGCAGGGCTTCATGGGCAATTACCTGGAGCGCAGCATGCAGGTGTTCCTGGATCAGCAGCAGCAGTTCCGTCAGCAGATGGGCAACCTGCTCGGGCAGACCCCGTGGGCGATGATGAACCAGCTGACCGAGCGCAACCTGGAGTTGTGGCAGGAGTTCCAGCGCAACTTCGGCGCCGGCTTCGGCCGCCCGGGTGGCCCGGGCACACCGCCGAGCCCGCCTGGGTCGAGCGGCCTGGGCAGCGGCCCGATGGGGACCGGCACGCATGGCTCGGCCGGTGGCAATCACGGCACGACCGGCAAGGCCCGCAACCGCGGCTAA

>XcviCFBP7111

TTGGCTGTTGCAAGCGCTGCTGCGGTGCGCCATCCTGCGCGCACTATCAGTGTGGGTGAACGTTCCATGGCCGGACTTCGCATCATCAAGAAGTATCCCAATCGCCGTCTCTACGACACGGAAATCTCCAGCTATATCACCATCGAAGATGTGCGCCAGTTGATCATCGATGGCGAAGAATTCGAAGTACGCGACGCCAAGAGCGGCGAGGACCTCAGCCGCGCAGTCCTGCTGCAAATCATCGCCGACCGCGAGCAGGACGGCGAGCCGATGCTCTCCACCCAGTTGCTGAGCCAGATCATCCGGTTTTACGGCGACTCGCTGCAGGGCTTCATGGGCAATTACCTGGAGCGCAGCATGCAGGTGTTCCTGGATCAGCAGCAGCAGTTCCGCCAGCAGATGGGTAACCTGCTCGGGCAGACCCCGTGGGCGATGATGAACCAATTGACCGAGCGCAACCTGGAGTTGTGGCAGGAGTTCCAGCGCAACTTCGGCGCCGGCTTCGGTCGTCCCGGCGGCCCTGGCACGCCGCCGAGCCCACCTGGGGCGAGCGGCCTGGGCAGCGGCCCGATGGGAACCGGCACGCATGGCTCGGCCGGTGGCAATCACGGCACGACCGGCAAAGCCCGCAACCGCGGCTAA

>XppCFBP6988

TTGGCTGTTGCAAGCGCTGCTGCGGTGCGCCATCCTGCGCGCACTATCAGTGTGGGTGAACGTTCCATGGCCGGACTTCGCATCATCAAGAAGTATCCCAATCGCCGTCTCTACGACACGGAAATCTCCAGCTACATCACCATCGAAGATGTGCGCCAGTTGATCATCGATGGCGAAGAATTCGAAGTACGCGACGCCAAGAGCGGCGAGGACCTCAGCCGCGCAGTCCTGCTGCAAATCATCGCCGACCGCGAGCAGGACGGCGAGCCGATGCTCTCCACCCAGTTGCTGAGCCAGATCATCCGGTTTTACGGCGACTCGCTGCAGGGCTTCATGGGCAATTACCTGGAGCGCAGCATGCAGGTGTTCCTGGATCAGCAGCAGCAGTTCCGCCAGCAGATGGGTAACCTGCTCGGGCAGACCCCGTGGGCGATGATGAACCAATTGACCGAGCGCAACCTGGAGTTGTGGCAGGAGTTCCAGCGCAACTTCGGCGCCGGCTTCGGTCGTCCCGGCGGCCCTGGCACGCCGCCGAGCCCACCTGGGGCGAGCGGCCTGGGCAGCGGCCCGATGGGAACCGGCACGCATGGCTCGGCCGGTGGCAATCACGGCACGACCGGCAAAGCCCGCAACCGCGGCTAA

>Xcf4834-R

TTGGCTGTTGCAAGCGCTGCTGCGGTGCGCCATCCTGCGCGCACTATCAGTGTGGGTGAACGTTCCATGGCCGGACTTCGCATCATCAAGAAGTATCCCAATCGCCGTCTCTACGACACGGAAATCTCCAGCTACATCACCATCGAAGATGTGCGCCAGTTGATCATCGATGGCGAAGAATTCGAAGTACGCGACGCCAAGAGCGGCGAGGACCTCAGCCGCGCAGTCCTGCTGCAAATCATCGCCGACCGCGAGCAGGACGGCGAGCCGATGCTCTCCACCCAGTTGCTGAGCCAGATCATCCGGTTTTACGGCGACTCGCTGCAGGGCTTCATGGGCAATTACCTGGAGCGCAGCATGCAGGTGTTCCTGGATCAGCAGCAGCAGTTCCGCCAGCAGATGGGTAACCTGCTCGGGCAGACCCCGTGGGCGATGATGAACCAATTGACCGAGCGCAACCTGGAGTTGTGGCAGGAGTTCCAGCGCAACTTCGGCGCCGGCTTCGGTCGTCCCGGCGGCCCTGGCACGCCGCCGAGCCCACCTGGGGCGAGCGGCCTGGGCAGCGGCCCGATGGGAACAGGCACGCATGGCTCGGCCGGTGGCAATCACGGCACGACCGGCAAAGCCCGCAACCGCGGCTAA

>Xca1566

TTGGCTGTTGCAAGCGCTGCTGCGGTGCGCCATCCTGCGCGCACTATCAGTGTGGGTGAACGTTCCATGGCCGGACTTCGCATCATCAAGAAGTATCCCAATCGCCGTCTCTACGACACGGAAATCTCCAGCTACATCACCATCGAAGATGTGCGCCAGTTGATCATCGATGGCGAAGAATTCGAAGTACGCGACGCCAAGAGCGGCGAGGACCTCAGCCGCGCAGTCCTACTGCAAATCATCGCCGACCGCGAGCAGGACGGCGAGCCGATGCTCTCCACCCAGTTGCTGAGCCAGATCATCCGGTTTTACGGCGACTCGCTGCAGGGCTTCATGGGCAATTACCTGGAGCGCAGCATGCAGGTGTTCCTGGATCAGCAGCAGCAGTTCCGCCAGCAGATGGGTAACCTGCTCGGGCAGACCCCGTGGGCGATGATGAACCAATTGACCGAGCGCAACCTGGAGTTGTGGCAGGAGTTCCAGCGCAACTTCGGCGCCGGCTTCGGCCGTCCCGGCGGCCCTGGCACGCCGCCGAGCCCACCTGGGGCGAGCGGCCTGGGCAGCGGCCCGATGGGAACCGGCACGCATGGCTCGGCCGGTGGCAATCACGGCACGACCGGCAAAGCCCGCAACCGCGGCTAA
