# Supplementary material for: Mutagenesis of PhaR, a Regulator Gene of Polyhydroxyalkanoate Biosynthesis of Xanthomonas oryzae pv. oryzae Caused Pleiotropic Phenotype Changes
Source: Front Microbiol. 2018 Dec 17;9:3046. doi: 10.3389/fmicb.2018.03046 (PMC6304360; doi:10.3389/fmicb.2018.03046)
Supplement: Supplementary file 2 [file Table_2.DOCX]

**Supplementary Data**

**Supplementary Tables**

**Table S1.** Strains and plasmids used in this study

| Designation | Relevant characteristics | Reference Sources |
| --- | --- | --- |
| Strains |  |  |
| *Xanthomonas* *oryzae* pv. *oryzae* |  |  |
| PXO99^A^ | Philippine race 6; azacytidine resistant clone of PXO99 | Lab collection |
| PXO99ΔPhaR | PXO99^A^ with the knockout of *PhaR* (Km^r^) | Lab collection |
| C-ΔPhaR | PXO99Δ*PhaR* complemened with pHM1*PhaR* (Km^r^ , Sp^r^) | This study |
| Plasmids |  |  |
| pA254 | Cosmid clone of PXO99^A^ containing *PhaR* gene | This study |
| pKD13 | Kanamycin resistance gene containing plasmid (Km^r^) | Lab collection |
| pPhaR*-*KD | Kanamycin gene flanked by *PhaR*_PXO99_ upstream and downstream fragments (Km^r^ , Ap^r^) | This study |
| pHM1 | Broad host range vector (Sp^r^) | Lab collection |
| pHM1*PhaR* | *PhaR*_PXO99_ gene cloned in pHM1 (Sp^r^) | This study |

**Table S2.** Primers used in this study

| **gene** | **Sequence (Forward primer/Reverse primer, 5’ → 3’ )** | **Product (bp)** |
| --- | --- | --- |
| ***KD*13** | ACGTCTTGAGCGATTGTGTA/AGTGATTGCGCCTACCCGGA | 1408 |
| ***PhaR*F1** | ATCGCCCTCAGCCTTACTTG/ AATATCCGGGTAGGCGCAATCACTAACGCCTGATTTGCCTAAGC | 893 |
| ***PhaR*F2** | AGCCTACACAATCGCTCAAGACGTAAGAGCGGCTAACAAAACGTCGC/ AGCCGTTCAAGCCGTGGTTC | 729 |
| ***PhaR***  ***PhaC***  ***PhaE***  ***PhaZ*** | TAAGCTTTTGGCTGTTGCAAGCGC/ GTACCTTAGCCGCGGTTGCGGGCCT  AAGTGGCGGGAGGAAAAC/TGCTCGCTCAGCTTAGACG  ACGGCAAGGTGGTGCTGTAT/CGGCATAGCACAAGGAAAACG  CGCATCGGCAGTTCTACG/CGCCTTCCACCACCAAAT | 656  191  330  333 |
| ***Hpa1*** | GACTTGGCTTCGCGATACAGG/ GGTCAAGATTCGACTGACCCG | 237 |
| ***16S rDNA*** | ACACAATTCGGCGGCAGCAC/TGACCCTTACCCTCCTCAGCATTT | 176 |
|  |  |  |
